# Proteomic analysis of plasma membranes isolated from undifferentiated and differentiated HepaRG cells

**DOI:** 10.1186/1477-5956-10-47

**Published:** 2012-08-02

**Authors:** Izabela Sokolowska, Cristina Dorobantu, Alisa G Woods, Alina Macovei, Norica Branza-Nichita, Costel C Darie

**Affiliations:** 1 Biochemistry & Proteomics Group, Department of Chemistry & Biomolecular Science, Clarkson University, 8 Clarkson Avenue, Potsdam, NY, 13699-5810, USA; 2 Department of Glycoproteins, Institute of Biochemistry of the Romanian Academy, Splaiul Independentei, 296, Sector 6, Bucharest, 060031, Romania

**Keywords:** Hepatocytes, HBV, Proteomics, Mass spectrometry, Differentiation

## Abstract

Liver infection with hepatitis B virus (HBV), a DNA virus of the *Hepadnaviridae* family, leads to severe disease, such as fibrosis, cirrhosis and hepatocellular carcinoma. The early steps of the viral life cycle are largely obscure and the host cell plasma membrane receptors are not known. HepaRG is the only proliferating cell line supporting HBV infection *in vitro*, following specific differentiation, allowing for investigation of new host host-cell factors involved in viral entry, within a more robust and reproducible environment. Viral infection generally begins with receptor recognition at the host cell surface, following highly specific cell-virus interactions. Most of these interactions are expected to take place at the plasma membrane of the HepaRG cells. In the present study, we used this cell line to explore changes between the plasma membrane of undifferentiated (−) and differentiated (+) cells and to identify differentially-regulated proteins or signaling networks that might potentially be involved in HBV entry. Our initial study identified a series of proteins that are differentially expressed in the plasma membrane of (−) and (+) cells and are good candidates for potential cell-virus interactions. To our knowledge, this is the first study using functional proteomics to study plasma membrane proteins from HepaRG cells, providing a platform for future experiments that will allow us to understand the cell-virus interaction and mechanism of HBV viral infection.

## Background

The hepatitis B virus (HBV) is a noncytopathic, hepatotropic DNA virus of the Hepadnaviridae family [1]. Infection with this virus leads to severe liver damage, such as fibrosis, cirrhosis and hepatocellular carcinoma [2]. Despite the existence of an efficient vaccine, more than 350 million people are currently HBV carriers at risk for developing life-threatening diseases.

While our understanding of HBV replication and assembly has advanced considerably in the last years, the early steps of the viral life cycle are still a matter of debate. This is mainly a consequence of the poor *in vitro* infectivity systems available, which until recently were based on primary human and chimpanzee hepatocytes [3]. Their accessibility is limited and the level of HBV replication is low, which makes the experimental data often difficult to interpret. The development of the HepaRG cell line, the only proliferating cells able to support the full HBV life cycle (following a specific differentiation treatment), unfolded new opportunities to investigate HBV infection in a more reproducible and reliable manner [4]. The ability of HepaRG to allow for HBV infection is reached only when cells are maintained quiescent at confluence and are treated with DMSO and hydrocortisone. While confluence alone is sufficient to activate many hepatic functions, DMSO treatment is compulsory for HBV productive infection. During differentiation, HepaRG cells express various liver functions in amounts comparable to those existing in primary hepatocytes [5-7]. Quantification of RNA levels within the whole population of differentiated cells showed high expression of adult hepatocytes-specific markers, such as albumin and aldolase B mRNAs, while the detoxification enzymes cytochrome P450, CYP 2E1 and CYP 3A4 were up-regulated in cells undergoing trabecular organization.

Generally, viral infection begins with receptor recognition and attachment to the host cell surface, followed by internalization of the virion by direct fusion at the plasma membrane, or endocytosis and later release from the endocytic vesicle. HBV appears to enter the target cells by receptor-mediated endocytosis, a process dependent on functional caveolin-1 expression [8]. Despite several potential cellular binding partners being reported to play a role in viral entry [4], none of these molecules was further confirmed to be the specific HBV receptor(s).

The rapid development of proteomics techniques has enabled the assessment of cellular proteins biosynthesis at a global scale, as well as the investigation of expression profile alterations under certain physiological or non-physiological conditions, with potential implications in cell function [9-11]. A previous proteomics study using HBV-uninfected and HBV-infected HepaRG cells identified 19 differentially-regulated proteins [12]. However, additional proteomic studies, more focused on plasma membrane proteins, (the first recognition partners during cell-virus interaction), are needed.

In the present study, we used the HepaRG cells to explore changes between the plasma membranes of undifferentiated (−) and differentiated (+) cells, and further identify differentially-regulated proteins that may potentially be involved in HBV entry or functional signaling networks that are activated upon cell-virus interaction. Our study identified a series of plasma-membrane-specific proteins, differentially expressed in (−) and (+) cells, with a potential role in viral infection. To our knowledge, this is the first study that focused on plasma membrane proteins from HePaRG cells using functional proteomics. The results obtained provide a platform for future investigations that will allow us to understand HBV cell-virus interactions and the molecular mechanisms of viral infection.

## Results & discussion

### Purification and verification of plasma membranes

**Figure 1:**
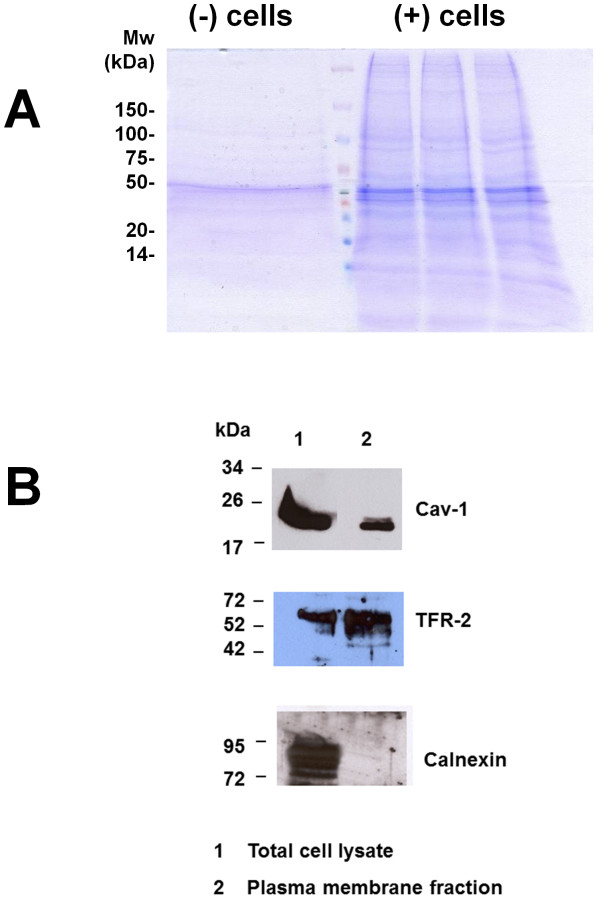
**SDS-PAGE of the proteins from the plasma membranes isolated from the undifferentiated (−) and differentiated HepaRG cells. A**: Coomassie stain of the SDS-PAGE gel showing the protein pattern for the plasma membrane of (−) and (+) cells. **B**: Expression of Cav-1, TRF-2 and calnexin in cell lysates (1) and plasma membrane fraction (2) of (−) cells was detected by Western blotting using the corresponding Abs. The molecular weight markers are indicated.

Upon purification, we separated the plasma membranes from the (−) cells and (+) cells by SDS-PAGE, stained them by Coomassie dye and visually compared the protein pattern between the plasma membrane preparations from (−) and (+) cells. As observed, there is a clear difference between the protein patterns in these two preparations (Figure 1A). A difference in the intensity of the Coomassie-stained bands was also observed between (−) and (+) samples, despite an equal number of cells being used for plasma membrane preparation. Most probably this is a result of a better extraction of the transmembrane proteins from differentiated cells, as a consequence of an increased plasma membrane fluidity during prolonged treatment with 1.8% DMSO. This behavior is not unusual and was also observed during extraction of lipid raft proteins from differentiated HepaRG cells (data not shown) and is not directly related to the differentiation process.

To confirm the plasma membrane isolation, total cell lysates, as well as a fraction of the (−) sample, were separated by SDS-PAGE and further analyzed by WB using antibodies against proteins with known plasma membrane or intracellular organelles localization. As observed in Figure 1B, expression of caveolin-1 (Cav-1) and transferrin receptor-2 (TRF-2) was detected in both, cell lysates and plasma membrane fraction, while the endoplasmic reticulum (ER) transmembrane protein, calnexin, was absent in the latter.

The latest investigations on HepaRG show that the number of differentiated cells, following DMSO treatment, is reasonably high (>50% of the total cell population) [13]. The significant up-regulation of hepatocyte-specific markers, considering the whole cell population, was clearly possible, ever since the cell line was described [4]. Thus, it is conceivable that changes of the level of expression (or post-translational modifications) of other proteins can be monitored in these cells.

### LC-MS/MS identification of plasma membrane proteins

**Figure 2:**
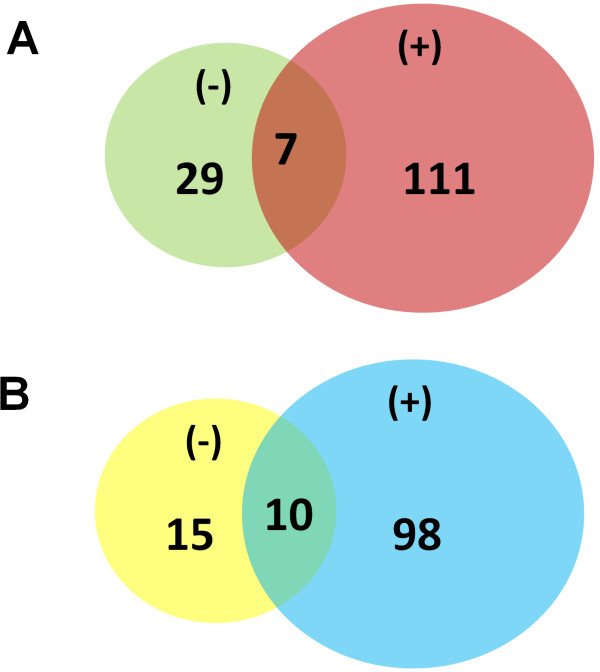
**Proteomic analysis of the proteins from (−) and (+) cells.** The Coomassie-stained SDS-PAGE gel (as the one shown in Figure 1A) was divided in gel pieces and then subjected to LC-MS/MS analysis, as described in the methods section. Venn diagrams in **A** &**B** show the number of proteins identified per condition (from both (−) and (+) cells), as well as the number of proteins that are common or different in the (−) and (+) cells. Here we show the results from experiment 1 in **A** and experiment 2 in **B**.

To further identify the proteins from the plasma membranes of the (−) and (+) cells, we cut bands out of the gel, digested them with trypsin and then analyzed them by LC/MS/MS. We performed two independent experiments, from two different preparations. Overall, we identified more proteins in the plasma membranes of the (+) cells, compared with the (−) cells. The results were consistent in both experiments. The outcome of two independent experiments is shown in Figure 2. Here are presented only the proteins identified with a Mascot score higher than 40. Also, the unnamed protein products, keratins and structural proteins (actin, tubulin) were removed from the final number of proteins presented in Figure 2. In experiment 1, we identified 118 proteins in the plasma membranes from (+) cells and 36 proteins in the plasma membranes from (−) cells. In this experiment, there was very little overlap between the two conditions (7 proteins). Similar results were observed in experiment 2: we identified 108 proteins in the plasma membranes from (+) cells and 25 proteins in the plasma membranes from (−) cells. The overlap between the two conditions was 10 proteins. The differentially identified proteins (proteins found only (−) but not in (+) and vice-versa) are presented in Tables 1 and 2. The complete lists with the proteins identified in our experiments are summarized in Additional file 1: Table S1 and Additional file 2: Table S2.

**Table 1:** List with the proteins that were differentially identified by LC-MS/MS analysis of the plasma membranes of undifferentiated (−) and differentiated (+) cells (experiment #1)

	**Proteins identified only in (-) cells**
**Protein Accession #**	**Protein_description**
gi|28614	aldolase A [Homo sapiens]
gi|134665	RecName: Full=superoxidase dismutase [Mn], mitochondrial, Flags: prescursor
gi|179930	carboxylesterase [Homo sapiens]
gi|2906146	malate dehydrogenase precursor [Homo sapiens]
gi|31645	glyceraldehyde-3-phosphate dehydrogenase [Homo sapiens]
gi|205830271	RecName: Full=Putative annexin A2-like protein; AltName: Full=Annexin A2 pseudogene 2
gi|30841303	manganese-containing superoxide dismutase [Homo sapiens]
gi|40068518	6-phosphogluconate dehydrogenase, decarboxylating [Homo sapiens]
gi|15012080	ACAT1 protein - Acetyl-CoA acetyltransferase [Homo sapiens]
gi|1723158	dihydrodiol dehydrogenase/bile acid-binding protein [Homo sapiens]
gi|134133226	POTE ankyrin domain family member E [Homo sapiens]
gi|4502013	adenylate kinase 2, mitochondrial isoform a [Homo sapiens]
gi|312137	fructose bisphosphate aldolase [Homo sapiens]
gi|5802974	thioredoxin-dependent peroxide reductase, mitochondrial isoform a precursor [Homo sapiens]
gi|257476	glutathione S-transferase A2 subunit [Homo sapiens]
gi|809185	Chain A, The Effect Of Metal Binding On The Structure Of Annexin V And Implications For Membrane Binding
gi|17389815	Triosephosphate isomerase 1 [Homo sapiens]
gi|511635	delta3, delta2-enoyl-CoA isomerase [Homo sapiens]
gi|56554357	Chain A, Binary Structure Of Human Decr Solved By Semet Sad.
gi|148733138	ubiquitously transcribed tetratricopeptide repeat protein Y-linked transcript variant 35 [Homo sapiens]
	**Proteins identified only in (+) cells**
gi|30749518	Chain A, Crystal Structure Of Human Liver Carboxylesterase In Complex With Tacrine
gi|13937981	Peptidylprolyl isomerase A (cyclophilin A) [Homo sapiens]
gi|37267	transketolase [Homo sapiens]
gi|693933	2-phosphopyruvate-hydratase alpha-enolase [Homo sapiens]
gi|2981743	Chain A, Secypa Complexed With Hagpia (Pseudo-Symmetric Monomer)
gi|35505	pyruvate kinase [Homo sapiens]
gi|31645	glyceraldehyde-3-phosphate dehydrogenase [Homo sapiens]
gi|1633054	Chain A, Cyclophilin A Complexed With Dipeptide Gly-Pro
gi|860986	protein disulfide isomerase [Homo sapiens]
gi|291463384	Chain K, Acetyl-Cypa:cyclosporine Complex
gi|4557305	fructose-bisphosphate aldolase A isoform 1 [Homo sapiens]
gi|6009628	brain carboxylesterase hBr3 [Homo sapiens]
gi|999892	Chain A, Crystal Structure Of Recombinant Human Triosephosphate Isomerase At 2.8 Angstroms Resolution.
gi|306891	90kDa heat shock protein [Homo sapiens]
gi|4505591	peroxiredoxin-1 [Homo sapiens]
gi|61656603	Heat shock protein HSP 90 -alpha 2
gi|55959290	annexin A1 [Homo sapiens]
gi|1710248	protein disulfide isomerase-related protein 5 [Homo sapiens]
gi|5729877	heat shock cognate 71 kDa protein isoform 1 [Homo sapiens]
gi|62088648	tumor rejection antigen (gp96) 1 variant [Homo sapiens]
gi|4505763	phosphoglycerate kinase 1 [Homo sapiens]
gi|386758	GRP78 precursor [Homo sapiens]
gi|2906146	malate dehydrogenase precursor [Homo sapiens]
gi|34709	manganese superoxide dismutase (MnSOD) [Homo sapiens]
gi|32189394	ATP synthase subunit beta, mitochondrial precursor [Homo sapiens]
gi|4505753	phosphoglycerate mutase 1 [Homo sapiens]
gi|21361176	retinal dehydrogenase 1 [Homo sapiens]
gi|438069	thiol-specific antioxidant protein [Homo sapiens]
gi|178375	aldehyde dehydrogenase [Homo sapiens]
gi|930063	neurone-specific enolase [Homo sapiens]
gi|5453549	peroxiredoxin-4 precursor [Homo sapiens]
gi|11128019	cytochrome c [Homo sapiens]
gi|458470	carboxylesterase [Homo sapiens]
gi|1065111	Chain A, Nmr Structure Of Mixed Disulfide Intermediate Between Mutant Human Thioredoxin And A 13 amino acid Peptide
	Comprising Its Target Site In Human Nfkb
gi|312137	fructose bisphosphate aldolase [Homo sapiens]
gi|913159	neuropolypeptide h3 [human, brain, Peptide, 186 aa]
gi|189238	neuroleukin [Homo sapiens]
gi|38648667	Fatty acid synthase [Homo sapiens]
gi|4503143	cathepsin D preproprotein [Homo sapiens]
gi|1065322	Chain A, A Surface Mutant (G82r) Of A Human Alpha-Glutathione S- Transferase
gi|6729803	Chain A, Heat-Shock 70kd Protein 42kd Atpase N-Terminal Domain
gi|189617	protein PP4-X [Homo sapiens]
gi|4758304	protein disulfide-isomerase A4 precursor [Homo sapiens]
gi|1040689	Human Diff6,H5,CDC10 homologue [Homo sapiens]
gi|544759	biliverdin-IX beta reductase isozyme I {EC 1.3.1.24} [human, liver, Peptide, 204 aa]
gi|62897129	heat shock 70kDa protein 8 isoform 1 variant [Homo sapiens]
gi|556516	dihydrodiol dehydrogenase isoform DD1 [Homo sapiens]
gi|1421609	Chain A, X-Ray Structure Of Nm23 Human Nucleoside Diphosphate Kinase B Complexed With Gdp At 2 Angstroms Resolution
gi|20070125	protein disulfide-isomerase precursor [Homo sapiens]
gi|729433	RecName: Full=Protein disulfide-isomerase A3; AltName: Full=58 kDa glucose-regulated protein; AltName:
gi|4757900	calreticulin precursor [Homo sapiens]
gi|5031857	L-lactate dehydrogenase A chain isoform 1 [Homo sapiens]
gi|1082886	tumor necrosis factor type 1 receptor associated protein TRAP-1 - human
gi|18204869	TUBA1B protein [Homo sapiens]
gi|5802966	destrin isoform a [Homo sapiens]
gi|119602577	plectin 1, intermediate filament binding protein 500kDa, isoform CRA_b [Homo sapiens]
gi|41322916	plectin isoform 1 [Homo sapiens]
gi|5174539	malate dehydrogenase, cytoplasmic isoform 2 [Homo sapiens]
gi|4503483	elongation factor 2 [Homo sapiens]
gi|3212355	Chain A, P11 (S100a10), Ligand Of Annexin Ii
gi|188492	heat shock-induced protein [Homo sapiens]
gi|21465695	Chain A, Human 3alpha-Hsd Type 3 In Ternary Complex With Nadp And Testosterone
gi|179930	carboxylesterase [Homo sapiens]
gi|4758012	clathrin heavy chain 1 [Homo sapiens]
gi|35830	ubiquitin activating enzyme E1 [Homo sapiens]
gi|4507953	14-3-3 protein zeta/delta [Homo sapiens]
gi|662841	heat shock protein 27 [Homo sapiens]
gi|4505257	moesin [Homo sapiens]
gi|4507357	transgelin-2 [Homo sapiens]
gi|4506467	radixin [Homo sapiens]
gi|6457378	cytovillin 2 [Homo sapiens]
gi|4505751	profilin-2 isoform b [Homo sapiens]
gi|6063147	ezrin [Homo sapiens]
gi|61104911	heat shock protein 90Bb [Homo sapiens]
gi|325533983	Chain A, Crystal Structure Of The Globular Domain Of Human Calreticulin
gi|119590453	EDAR-associated death domain, isoform CRA_a [Homo sapiens]
gi|311772317	Chain A, Crystal Structure Of Human Nad Kinase
gi|4504067	aspartate aminotransferase, cytoplasmic [Homo sapiens]
gi|5630077	similar to ALR; similar to AAC51735 (PID:g2358287) [Homo sapiens]
gi|896473	iron-responsive regulatory protein/iron regulatory protein 1 [Homo sapiens]
gi|5803225	14-3-3 protein epsilon [Homo sapiens]
gi|4826643	annexin A3 [Homo sapiens]
gi|332841153	PREDICTED: heat shock protein 105 kDa isoform 1 [Pan troglodytes]
	**Proteins identified in(-) and (+) cells**
gi|4757756	annexin A2 isoform 2 [Homo sapiens]
gi|181250	cyclophilin [Homo sapiens]
gi|291463380	Chain B, Free Acetyl-Cypa Orthorhombic Form
gi|215273984	RecName: Full=Putative inactive carboxylesterase 4; AltName: Full=Inactive carboxylesterase 1 pseudogene 1
gi|809185	Chain A, The Effect Of Metal Binding On The Structure Of Annexin V And Implications For Membrane Binding
gi|4507913	wiskott-Aldrich syndrome protein family member 1
gi|438069	thiol-specific antioxidant protein [Homo sapiens]

**Table 2:** List with the proteins that were differentially identified by LC-MS/MS analysis of the plasma membranes of undifferentiated (−) and differentiated (+) cells (experiment #2)

	**Proteins identified only in (-) cells**
**Protein Accession #**	**Protein_description**
gi|458470	carboxylesterase [Homo sapiens]
gi|62088648	tumor rejection antigen (gp96) 1 variant [Homo sapiens]
gi|28614	aldolase A [Homo sapiens]
gi|18645167	Annexin A2 [Homo sapiens]
gi|31179	enolase [Homo sapiens]
gi|34707	Manganese superoxide dismutase [Homo sapiens]
gi|4502013	adenylate kinase 2, mitochondrial isoform a [Homo sapiens]
gi|178390	aldehyde dehydrogenase [Homo sapiens]
gi|4504505	peroxisomal multifunctional enzyme type 2 isoform 2 [Homo sapiens]
gi|4503285	aldo-keto reductase family 1 member C2 isoform 1 [Homo sapiens]
gi|1723158	dihydrodiol dehydrogenase/bile acid-binding protein [Homo sapiens]
gi|6009628	brain carboxylesterase hBr3 [Homo sapiens]
gi|386758	GRP78 precursor [Homo sapiens]
gi|4507913	wiskott-Aldrich syndrome protein family member
	**Proteins identified only in (+) cells**
gi|306891	90kDa heat shock protein [Homo sapiens]
gi|1633054	Chain A, Cyclophilin A Complexed With Dipeptide Gly-Pro
gi|4507677	endoplasmin precursor [Homo sapiens]
gi|75766275	Chain A, Crystal Structure Of Human Cypa Mutant K131a
gi|61656603	Heat shock protein HSP 90 - alpha 2 [Homo sapiens]
gi|179930	carboxylesterase [Homo sapiens]
gi|2981743	Chain A, Secypa Complexed With Hagpia (Pseudo-Symmetric Monomer)
gi|4757756	annexin A2 isoform 2 [Homo sapiens]
gi|4826898	profilin-1 [Homo sapiens]
gi|342350777	Chain A, Human Annexin V With Incorporated Methionine Analogue Azidohomoalanine
gi|32189394	ATP synthase subunit beta, mitochondrial precursor [Homo sapiens]
gi|157833780	Chain A, Human Annexin V With Proline Substitution By Thioproline
gi|53791219	filamin A [Homo sapiens]
gi|31645	glyceraldehyde-3-phosphate dehydrogenase [Homo sapiens]
gi|14327942	HSP90B1 protein [Homo sapiens]
gi|28614	aldolase A [Homo sapiens]
gi|662841	heat shock protein 27 [Homo sapiens]
gi|999892	Chain A, Crystal Structure Of Recombinant Human Triosephosphate Isomerase At 2.8 Angstroms
	Resolution.
gi|35505	pyruvate kinase [Homo sapiens]
gi|693933	2-phosphopyruvate-hydratase alpha-enolase [Homo sapiens]
gi|4503483	elongation factor 2 [Homo sapiens]
gi|5729877	heat shock cognate 71 kDa protein isoform 1 [Homo sapiens]
gi|4505591	peroxiredoxin-1 [Homo sapiens]
gi|6525069	tumor necrosis factor type 1 receptor associated protein [Homo sapiens]
gi|4502101	annexin A1 [Homo sapiens]
gi|189617	protein PP4-X [Homo sapiens]
gi|4758012	clathrin heavy chain 1 [Homo sapiens]
gi|34419635	70kDa heat shock protein 6 [Homo sapiens]
gi|4507357	transgelin-2 [Homo sapiens]
gi|577295	KIAA0088 [Homo sapiens]
gi|35830	ubiquitin activating enzyme E1 [Homo sapiens]
gi|6470150	BiP protein [Homo sapiens]
gi|5453790	nicotinamide N-methyltransferase [Homo sapiens]
gi|5803227	14-3-3 protein theta [Homo sapiens]
gi|38648667	Fatty acid synthase [Homo sapiens]
gi|31746	glutathione-insulin transhydrogenase (216 AA) [Homo sapiens]
gi|1421609	Chain A, X-Ray Structure Of Nm23 Human Nucleoside Diphosphate Kinase B Complexed With
	Gdp At 2 Angstroms Resolution
gi|438069	thiol-specific antioxidant protein [Homo sapiens]
gi|61104911	heat shock protein 90Bb [Homo sapiens]
gi|4758304	protein disulfide-isomerase A4 precursor [Homo sapiens]
gi|5802974	thioredoxin-dependent peroxide reductase, mitochondrial isoform a precursor [Homo sapiens]
gi|3318841	Chain A, Horf6 A Novel Human Peroxidase Enzyme
gi|230867	Chain R, Twinning In Crystals Of Human Skeletal Muscle D- Glyceraldehyde-3-Phosphate
gi|230867	Dehydrogenase
gi|5031857	L-lactate dehydrogenase A chain isoform 1 [Homo sapiens]
gi|4507877	vinculin isoform VCL [Homo sapiens]
gi|1710248	protein disulfide isomerase-related protein 5 [Homo sapiens]
gi|5031635	cofilin-1 [Homo sapiens]
gi|83753119	Chain A, Crystal Structure Of Human Full-Length Vinculin (Residues 1- 1066)
gi|157879202	Chain B, Crystal Structures Of Native And Inhibited Forms Of Human Cathepsin D: Implications
	For Lysosomal Targeting And Drug Design
gi|74722493	RecName: Full=Putative heat shock protein HSP 90-alpha A4; AltName: Full=Heat shock 90 kDa
gi|74722493	protein 1 alpha-like 2; AltName: Full=Heat shock protein 90-alpha D
gi|181250	cyclophilin [Homo sapiens]
gi|182855	80K-H protein [Homo sapiens]
gi|37267	transketolase [Homo sapiens]
gi|1065111	Chain A, High Resolution Solution Nmr Structure Of Mixed Disulfide Intermediate Between
	Mutant Human Thioredoxin And A 13 Residue Peptide Comprising Its Target Site In Human Nfkb
gi|1477646	plectin [Homo sapiens]
gi|4507953	14-3-3 protein zeta/delta [Homo sapiens]
gi|2138314	lysyl hydroxylase isoform 2 [Homo sapiens]
gi|283807248	Chain A, Crystal Structure Analysis Of W21a Mutant Of Human Gsta1-1 In Complex With
	S-Hexylglutathione
gi|6005942	transitional endoplasmic reticulum ATPase [Homo sapiens]
gi|1065322	Chain A, A Surface Mutant (G82r) Of A Human Alpha-Glutathione S- Transferase Shows Decreased
gi|1065322	Thermal Stability And A New Mode Of Molecular Association In The Crystal
gi|799177	100 kDa coactivator [Homo sapiens]
gi|4505753	phosphoglycerate mutase 1 [Homo sapiens]
gi|5107666	Chain A, Structure Of Importin Beta Bound To The Ibb Domain Of Importin Alpha
gi|158937236	puromycin-sensitive aminopeptidase [Homo sapiens]
gi|5825506	fatty acid synthase/estrogen receptor fusion protein [Homo sapiens]
gi|5257007	beta-cop homolog [Homo sapiens]
gi|896473	iron-responsive regulatory protein/iron regulatory protein 1 [Homo sapiens]
gi|34707	Manganese superoxide dismutase [Homo sapiens]
gi|4506715	40S ribosomal protein S28 [Homo sapiens]
gi|3212355	Chain A, P11 (S100a10), Ligand Of Annexin Ii
gi|1065361	Chain A, Human Adp-Ribosylation Factor 1 Complexed With Gdp, Full Length Non-Myristoylated
gi|159162145	Chain A, Rotamer Strain As A Determinant Of Protein Structural Specificity
gi|51247357	Chain A, Crystal Structure Of A Multiple Hydrophobic Core Mutant Of Ubiquitin
gi|215273984	RecName: Full=Putative inactive carboxylesterase 4; AltName: Full=Inactive carboxylesterase 1
gi|215273984	pseudogene 1; AltName: Full=Placental carboxylesterase 3; Short=PCE-3; Flags: Precursor
gi|4502013	adenylate kinase 2, mitochondrial isoform a [Homo sapiens]
gi|5803013	endoplasmic reticulum resident protein 29 isoform 1 precursor [Homo sapiens]
gi|229532	ubiquitin
gi|31615803	Chain A, Synthetic Ubiquitin With Fluoro-Leu At 50 And 67
gi|2627129	polyubiquitin [Homo sapiens]
gi|6755368	40S ribosomal protein S18 [Mus musculus]
gi|313103963	Chain D, Structure And Control Of The Actin Regulatory Wave Complex
gi|6114601	stromal antigen 3, (STAG3) [Homo sapiens]
gi|4588526	nuclear chloride channel [Homo sapiens]
	**Proteins identified in(-) and (+) cells**
gi|32189394	ATP synthase subunit beta, mitochondrial precursor [Homo sapiens]
gi|31645	glyceraldehyde-3-phosphate dehydrogenase [Homo sapiens]
gi|1710248	protein disulfide isomerase-related protein 5 [Homo sapiens]
gi|215273984	RecName: Full=Putative inactive carboxylesterase 4; AltName: Full=Inactive carboxylesterase 1
gi|215273984	pseudogene 1; AltName: Full=Placental carboxylesterase 3; Short=PCE-3; Flags: Precursor
gi|4758304	protein disulfide-isomerase A4 precursor [Homo sapiens]
gi|4505591	peroxiredoxin-1 [Homo sapiens]
gi|303618	phospholipase C-alpha [Homo sapiens]
gi|5031857	L-lactate dehydrogenase A chain isoform 1 [Homo sapiens]
gi|5802974	thioredoxin-dependent peroxide reductase, mitochondrial isoform a precursor [Homo sapiens]
gi|306891	90kDa heat shock protein [Homo sapiens]

It has been documented that the plasma membrane is the entry point for viruses [14-16]. Therefore, we looked in our experiments for proteins that are possible interaction partners with viruses. One example is the Annexin family of proteins. For example, Annexin A2 (gi 4757756), a calcium-regulated protein that binds to the plasma membrane, is usually a heterotetramer of two Annexin A2 proteins and two S100A10 (gi 3212355) proteins. We identified both Annexin A2 proteins and S100A10 in the plasma membrane of the (+) cells, but not from the (−) cells, confirming the established interaction between these 2 proteins. Since Annexin A2 already has a history of interacting with viruses [17-19], this suggests that an interaction with HBV may well be possible.

**Figure 3:**
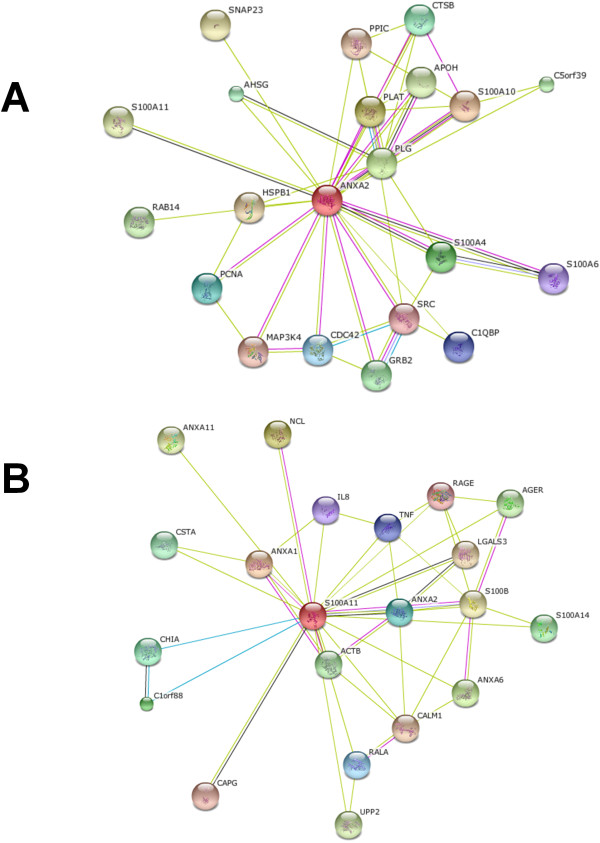
**Map of possible interaction partners for Annexin A2, Annexin A2 and S100A10. A**: Annexin A2 (ANXA2) is predicted to interact with S100A10 and form a heterotetramer. **B**: Annexin A1 is predicted to interact with Annexin A2, via S100A11, ACTB or via ILB and TNF.

**Figure 4:**
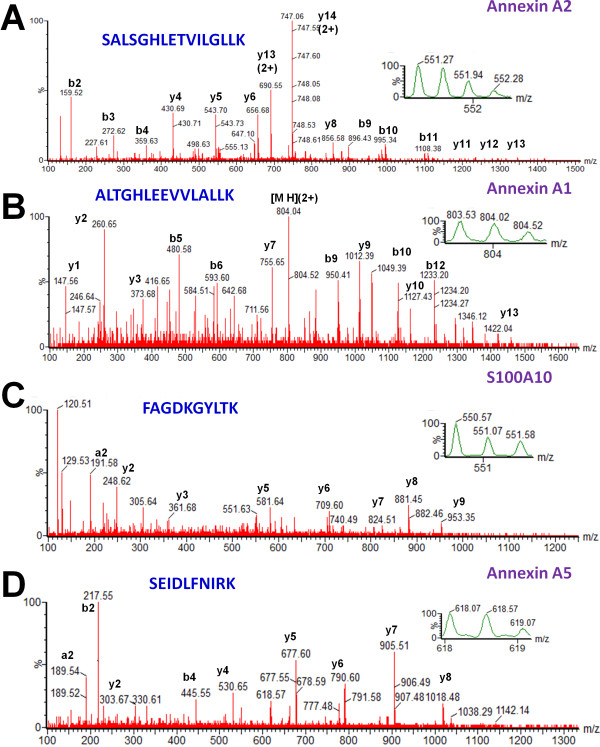
**MS/MS spectra of precursor ions that correspond to peptides that are part of Annexin A2, Annexin A1, S100A10, or Annexin A5.** (**A**) A triple-charged peak at m/z of 551.27 (expanded in the inbox) was fragmented by MS/MS and produced a MS/MS spectrum. Fragmentation of the peptide backbone in the MS/MS produced a series of b and y peaks, marked in the MS/MS. Data analysis of the b and y peaks from the MS/MS spectrum led to identification of a peptide with the sequence SALSGHLETVILGLLK, which was part of Annexin A2 protein. (**B**): A double-charged peak at m/z of 803.53 (expanded in the inbox) was fragmented by MS/MS and produced a MS/MS spectrum. Data analysis of the MS/MS spectrum led to identification of a peptide with the sequence ALTGHLEEVVLALLK, which was part of Annexin A1 protein. (**C**) A double-charged peak at m/z of 550.57 (expanded in the inbox) was fragmented by MS/MS and produced a MS/MS spectrum. Data analysis of this MS/MS spectrum led to identification of a peptide with the sequence FAGDKGYLTK, which was part of S100A10 protein. (**D**) A double-charged peak at m/z of 618.07 (expanded in the inbox) was fragmented by MS/MS and produced a MS/MS spectrum. Data analysis of this MS/MS spectrum led to identification of a peptide with the sequence SEIDLFNIRK, which was part of Annexin A5 protein.

Two other proteins, also from the Annexin family, identified in our experiments were Annexin A1 (gi 4502101) and Annexin A5 (gi 157833780). Annexin A1 is a known phospholipase A2 inhibitory protein, but is also predicted to interact with Annexin A2 and perhaps form a protein complex (Figure 3). However, Annexin A1 was identified only in the plasma membranes from (+) cells, but not from the (−) cells, suggesting that this protein may be specific for the plasma membrane of (+) cells. The other protein, Annexin A5 is not predicted to interact with any of the other Annexins. However, it is well documented that Annexin A5 is an interaction partner for HBV [20]. Examples of MSMS spectra that correspond to peptides that are part of Annexin A2, Annexin A1, S100A10 protein and Annexin A5 are shown in Figure 4.

To further confirm that Annexin 2 and S100A10 interact with each other and also to investigate the interaction partners of these proteins and of other Annexin proteins, we explored the protein-protein interactions using the Search Tool for the Retrieval of Interacting Genes (STRING), a software tool that identifies known (experimentally documented) and predicted (theoretical only and yet to be demonstrated by experiments) protein-protein interactions [21-24]. As observed, we did identify Annexin A2 as an interaction partner for S100A10 protein. However, we also identified a connection between Annexin A2 and Annexin A1, via S100A11, ACTB or via ILB and TNF proteins. The predicted interaction partners for Annexins A1 & A2 are presented in Figure 3.

### Differential distribution of the proteins from the plasma membranes of (−) and (+) cells

**Figure 5:**
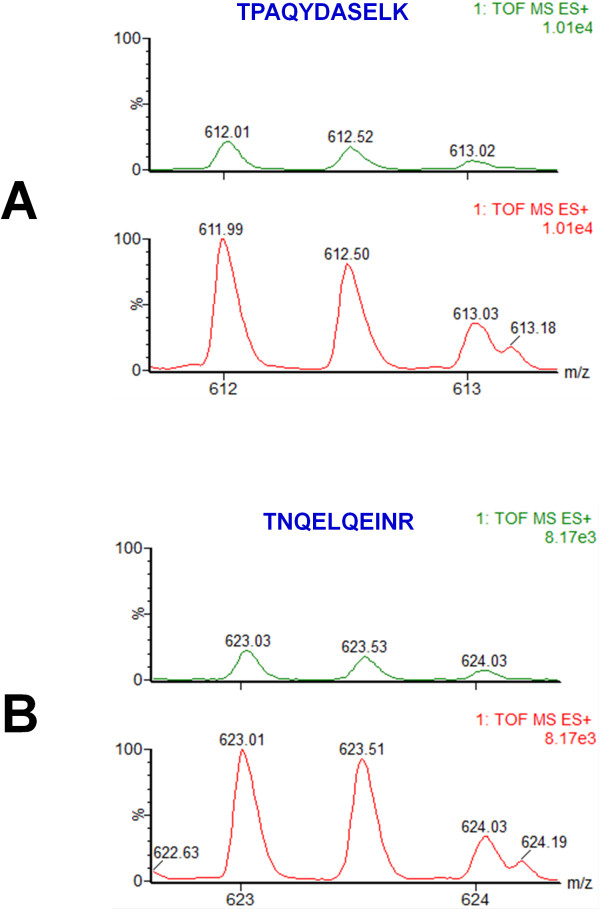
**Comparison of the intensities of MS spectra for peaks corresponding to peptides that are part of Annexin A2 protein from the plasma membranes of (−) and (+) cells, found by SDS-PAGE and MS. A**: The peak with m/z of 612.01 (2+) that corresponds to peptide TPAQYDASELK has a higher intensity in the plasma membranes of (+) cells, compared with the (−) ones. **B**: The peak with m/z of 623.03 (2+) that corresponds to peptide TNQELQEINR has a higher intensity in the plasma membranes of (+) cells, compared with the (−) ones. The intensity scale for the spectra from both (−) and (+) cells for each individual peptide was identical.

The differential distribution of the proteins found by SDS-PAGE and LC-MS/MS in the plasma membranes of both (−) and (+) cells was also evaluated by their relative abundance using label-free methods for relative quantitation. These proteins differed in their abundance by either an increase or decrease of their relative amounts, as determined by both Mascot score (Additional file 1: Table S1), emPAI score [25], or by comparison of the relative intensity of the precursor ions that correspond to peptides that were part of the same proteins and that were identified in both (−) and (+) cells. The relative quantitation of these proteins was mostly used to determine whether some proteins were indeed specific to the plasma membranes from (+) cells, but not (−) cells. Using Annexin proteins as example, we looked at both the Mascot scores and emPAI scores for these proteins, as well as for the number of peptides identified per protein per condition in the database search, as well as direct comparison of the intensities of the precursor ions that correspond to the same peptide and for which MS/MS was observed in the same protein in both (−) and (+) conditions. For example, in the plasma membranes of (+) cells we identified Annexin A2 isoform 2 based on the MS/MS of 22 peptide, a mascot score of 957 and an emPAI score of 3.01, as compared with identification of the same protein in the plasma membranes of (−) cells, where we identified the same protein based on the MS/MS of only 6 peptides, a mascot score of 213 and an emPAI score of 0.63. To confirm that the relative amounts of Annexin A2 in the plasma membranes from (+) cells, but not (−) cells are indeed different in these two conditions, as reflected by the number of peptide and by Mascot scores in each condition, we further compared the intensity of precursor ions for two different peptides that were part of the same Annexin A2 protein and that were identified in the plasma membrane of the (−) and (+) cells. The intensity scale for the spectra for these peaks was normalized to identical number of counts. This comparison is shown in Figure 5. As observed, for the peak with m/z of 612.01 (2+) that corresponds to a peptide with the amino acid sequence TPAQYDASELK, a higher amount of this peptide is observed in the plasma membranes from the (+) cells, compared with the (−) cells (Figure 5A). Similar results were also observed for the peak with m/z of 623.03 (2+) that corresponds to a peptide with the amino acid sequence TNQELQEINR (Figure 5B).

### Concluding remarks

In this study, we used the HepaRG cells to investigate the differences between the protein content of the plasma membranes from differentiated and undifferentiated cells. We aimed to identify functional signaling networks and plasma membrane molecules that are expressed in differentiated cells and may potentially be involved in HBV entry. Using a proteomics approach, we identified the differentially expressed proteins and also concluded that they may form protein complexes such as Annexin A2 and S100A10 protein heterotetramer, with potential implications in cell-virus interaction. This approach is not only a source of the proteins present in the plasma membranes of the (−) and (+) cells, but also a starting point for identification of post-translational modifications of these proteins, as well as for determination of stable and transient protein-protein interactions, specifically HepaRG cell - HBV proteins interactions.

### Experimental design

#### Chemicals

All chemicals were purchased from Sigma-Aldrich, unless mentioned otherwise**.**

#### Cell culture and differentiation

HepaRG cells (a kind gift from Dr. David Durantel, INSERM U871, Lyon, France) were grown in T75 flasks, in William’s E medium (Gibco) supplemented with 10% FCS, 50 units/ml penicillin, 50 μg/ml streptomycin, 2 mM GlutaMAX, 5 μg/ml insulin, and 5 x 10^5^ M hydrocortisone hemisuccinate, as described [4]. To induce differentiation, cells were maintained for 2 weeks in William’s complete medium, without splitting, followed by 2 weeks in the same medium containing 1.8% DMSO. The typical cell morphology associated with differentiation was constantly monitored under the microscope and the up-regulation of albumin and aldolase B mRNAs was confirmed at the end of the differentiation process.

#### Preparation of plasma membranes

Differentiated (+) and non-differentiated (−) HepaRG cells were amplified in T75cm^2^ flasks (6 for each condition). All further steps were performed at 4°C. Cells were washed twice with 5 ml buffer A (0.25 M sucrose, 1 mM EDTA and 20 mM Tricine, pH = 7.8) and gently scraped in 3 ml of the same buffer. The cells were pelleted by centrifugation at 1400xg for 5 min, resuspended in 1 ml buffer A and disrupted with 20 strokes in a Douncer homogenizer. The homogenate was centrifuged at 1000xg for 10 min. The post nuclear supernatant (PNS) was stored on ice, and the pellet was resuspended in 1 ml buffer A, re-homogenized with 20 strokes in a Douncer homogenizer, as above. The first and second PNS were combined and layered on top of a 23 ml of 30% Percoll in buffer A, followed by centrifugation at 28,000 rpm in a Beckman ultracentrifuge SW41Ti rotor, for 30 min. The plasma membrane fraction was visible as a ring at approximately 5.7 cm from the bottom of the tube. This was collected and the Percoll was removed by dilution in 9.5 ml cold phosphate buffer saline (PBS), followed by 2 h ultracentrifugation at 30,000 rpm, as above. The supernatant was concentrated on a 10 kDa cutt-of centricon (Millipore) to a final volume of 150 μl.

#### SDS-PAGE and Western blotting

The isolated membranes were solubilized in Laemmli sample buffer for 5 minutes at 95°C and separated by SDS-PAGE, followed by Coomassie blue staining. To validate the plasma membrane fraction purification, the proteins were also transferred to nitrocellulose membranes (GE Healthcare) using a semi-dry blotter (Millipore). The blots were incubated with goat anti-TFR-2, goat anti-Calnexin (both Santa Cruz Biotechnology, dilution 1/1000), or rabbit anti–Cav-1 (Cell Signaling Technology, dilution 1/1000) antibodies (Ab), respectively, followed by donkey anti-goat (Santa Cruz Biotechnology, dilution 1/2000) or goat anti-rabbit horseradish peroxidase (HRP) Ab (Pierce, dilution 1/1000). Proteins were detected using the ECL (GE Healthcare) or the SuperSignal West Femto maximum Sensitivity Substrate (Thermo Scientific) detection systems according to the manufacturers’ instructions

#### Protein digestion and peptide extraction

The Coomassie-stained SDS-PAGE gels were cut into 3 gel pieces for each condition (plasma membranes isolated from undifferentiated (−) and differentiated (+) cells), and then treated according to published protocols [26-29]. Briefly, the gel pieces were washed in high purity HPLC grade water for 20 minutes under moderate shaking and then and cut into very small pieces. The gel pieces were then dehydrated by incubation for 20 minutes in 50 mM ammonium bicarbonate, 20 minutes in 50 mM ammonium bicarbonate/50% acetonitrile, and 20 minutes in 100% acetonitrile. These three steps were performed under moderate shaking at room temperature. After the last incubation step, the gel pieces were dried in a Speed-vac concentrator and then rehydrated with 50 mM ammonium bicarbonate. The washing procedure was repeated twice. The dried gel bands were then rehydrated with a solution containing 10 mM DTT and 50 mM ammonium bicarbonate and incubated for 45 minutes at 56°C. DTT solution reduced the disulfide bridges in the proteins from the gel. The DTT solution was then replaced by a solution containing 100 mM iodoacetamide and 50 mM ammonium bicarbonate and further incubated for 45 minutes in the dark, with occasional vortexing. In this step, the cysteine residues were irreversibly modified by iodoacetamide to form carbamydomethyl-cysteine. The initial washing procedure was then repeated one more time, and then the gel pieces were dried in the Speed-vac concentrator and then rehydrated using 10 ng/μL trypsin (this leaves proteins at the peptide backbone at the Arg and Lys residues) in 50 mM ammonium bicarbonate, and then incubated overnight at 37°C under low shaking. The resulting peptides were extracted from the gel pieces by incubation with 5% formic acid/50 mM ammonium bicarbonate/50% acetonitrile (twice) and with 100% acetonitrile (once) under moderate shaking. Solutions containing peptide mixture were then combined and then dried in a Speed-vac concentrator. The peptides were then solubilized in 20 μL of 0.1% formic acid/2% acetonitrile/HPLC water, placed in UPLC vials and further used for LC-MS/MS analysis.

#### LC-MS/MS

The peptides mixture was analyzed by reversed phase liquid chromatography (LC) and MS (LC-MS/MS) using a NanoAcuity UPLC (Micromass/Waters, Milford, MA) coupled to a Q-TOF Micro MS (Micromass/Waters, Milford, MA), according to published procedures [29-31]. Briefly, the peptides were loaded onto a 100 μm x 10 mm nanoAquity BEH130 C18 1.7 μm UPLC column (Waters, Milford, MA) and eluted over a 60 minute gradient of 2–80% organic solvent (acetonitrile containing 0.1% formic acid) at a flow rate of 400 nL/min. The aqueous solvent was HPLC water containing 0.1% formic acid. The column was coupled to a Picotip Emitter Silicatip nano-electrospray needle (New Objective, Woburn, MA). MS data acquisition involved survey MS scans and automatic data dependent analysis (DDA) of the top three ions with the highest intensity ions with the charge of 2+, 3+ or 4+ ions. The MS/MS was triggered when the MS signal intensity exceeded 10 counts/second. In survey MS scans, the three most intense peaks were selected for collision-induced dissociation (CID) and fragmented until the total MS/MS ion counts reached 10,000 or for up to 6 seconds each. Preliminary experiments were performed using an Alliance 2695 HPLC (Waters Corp, Milford, MA) that was coupled to the same Q-TOF Micro MS (Micromass/Waters, Milford, MA) described above. The peptides mixture was loaded onto an XBridge^TM^ C18 3.5 μm, 2.1 x 100 mm column (Waters Corporation, Milford, MA) and eluted over a 60 minutes gradient of 2–100% acetonitrile containing 0.1% formic acid at a flow rate of 200 μL/min. The aqueous phase was HPLC water containing 0.1% formic acid. The MS parameters in these experiments were unchanged from the previously described settings, except the source (micro source instead of nanosource). The entire procedure used was previously described [29,32]. Calibration was performed for both precursor and product ions using either 1 pmol or 100 fmol GluFib standard peptide (Glu1-Fibrinopeptide B) with the sequence EGVNDNEEGFFSAR, with the monoisotopic m/z of 1770.68. The precursor ion monitored was the double charged peak of GluFib, with m/z of 785.84.

#### Data processing and protein identification

The raw data were processed using ProteinLynx Global Server (PLGS, version 2.4) software with the following parameters: background subtraction of polynomial order 5 adaptive with a threshold of 30%, two smoothings with a window of three channels in Savitzky-Golay mode and centroid calculation of top 80% of peaks based on a minimum peak width of 4 channels at half height. The resulting pkl files were submitted for database search and protein identification to the public Mascot database search ( http://www.matrixscience.com, Matrix Science, London, UK) using the following parameters: human databases from NCBI and SwissProt, parent mass error of 1.3 Da, product ion error of 0.8 Da, enzyme used: trypsin, one missed cleavage, and carbamidomethyl-Cysteine as fixed modification and Methionine oxidized as variable modification. To identify the false negative results, we used additional parameters such as different databases or organisms, a narrower error window for the parent mass error (1.2 and then 0.2 Da) and for the product ion error (0.6 Da), and up to two missed cleavage sites for trypsin. In addition, the pkl files were also searched against in-house PLGS database version 2.4 ( http://www.waters.com) using searching parameters similar to the ones used for Mascot search. The Mascot and PLGS database search provided a list of proteins for each gel band. To eliminate false positive results, for the proteins identified by either one peptide or a mascot score lower than 50, we verified the MS/MS spectra that led to identification of a protein. The proteins identified in our experiments are presented in Additional file 1: Table S1 and Additional file 2: Table S2. These proteins were identified with a Mascot score higher than 40. The proteins identified with a Mascot score lower than 40 were not considered, but the data can be provided upon request. The MS/MS spectra that allowed identification of a protein based on only one peptide are provided in Additional file 3: Figure S1. The MS/MS spectra provided in Additional file 3: Figure S1 were identified in Mascot database search with a score of 50 or higher in (+) cells. For the (−) cells, all MS/MS spectra are shown.

## Abbreviations

HepaRG cells: A hepatoma-derived cell line, HBV, hepatitis B virus; SDS-PAGE: Aodium dodecyl sulfate-polyacrylamide gel electrophoresis; WB: Western blotting; MS: Mass spectrometry; LC-MS/MS: Liquid chromatography mass spectrometry; TIC: Total ion current; m/z: Mass/charge; CID: Collision-induced dissociation.

## Competing interests

The authors declare that they have no competing interests.

## Authors’ contributions

IS, CD, NBN & CCD designed the study. IS, CD, AGW & AM performed the work. IS, CD, AGW, AM, NBN & CCD interpreted the data. IS, CD, AGW, AM, NBN & CCD wrote the manuscript. IS, CD AGW, AM, NBN & CCD approved the manuscript. All authors read and approved the final manuscript.

## Supplementary Material

Additional file 1 **Table S1. **LC/MS/MS experiment (experiment #1) of the proteins from the plasma membranes of undifferentiated (−) and differentiated (+) cells. Click here for file

Additional file 2 **Table S2.** LC/MS/MS experiment (experiment #2) of the proteins from the plasma membranes of undifferentiated (−) and differentiated (+) cells. Click here for file

Additional file 3 **Figure S1.** MS/MS spectra that led to identification of a protein by a unique peptide. In the (+) cells, the MS/MS spectra were identified with a Mascot score of 50 or higher. In the (−) cells, all MS/MS spectra are shown. Click here for file
