# Supplementary material for: Proteomic analysis of plasma membranes isolated from undifferentiated and differentiated HepaRG cells
Source: Proteome Sci. 2012 Aug 2;10:47. doi: 10.1186/1477-5956-10-47 (PMC3527237; doi:10.1186/1477-5956-10-47)

Supplemental Figure 1

MSMS spectra for the proteins identified by one peptide
Experiment 1, undifferentiated cells

IAAAILNTPDLR

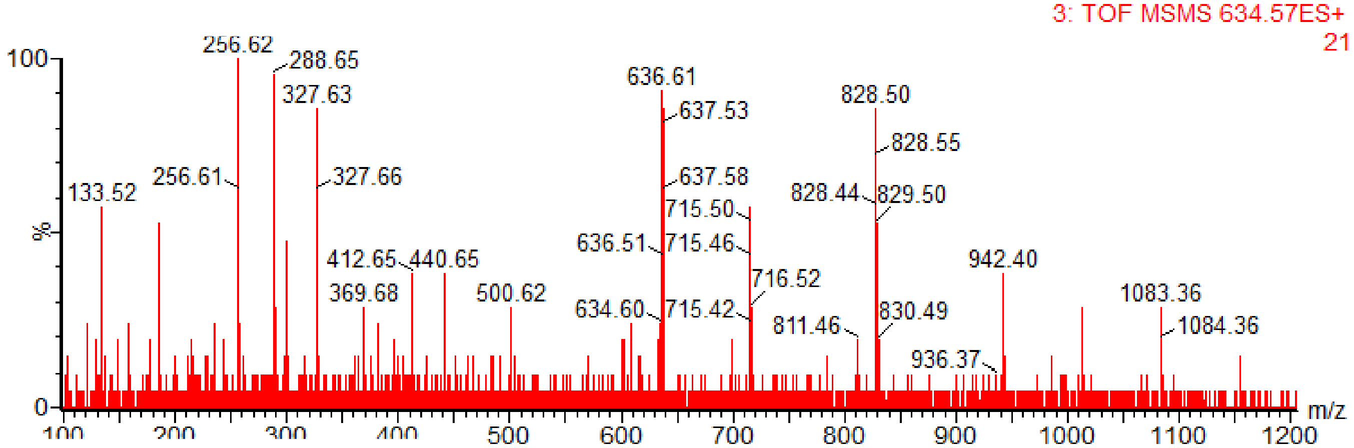

LLIVSNPVDILTYVAWK

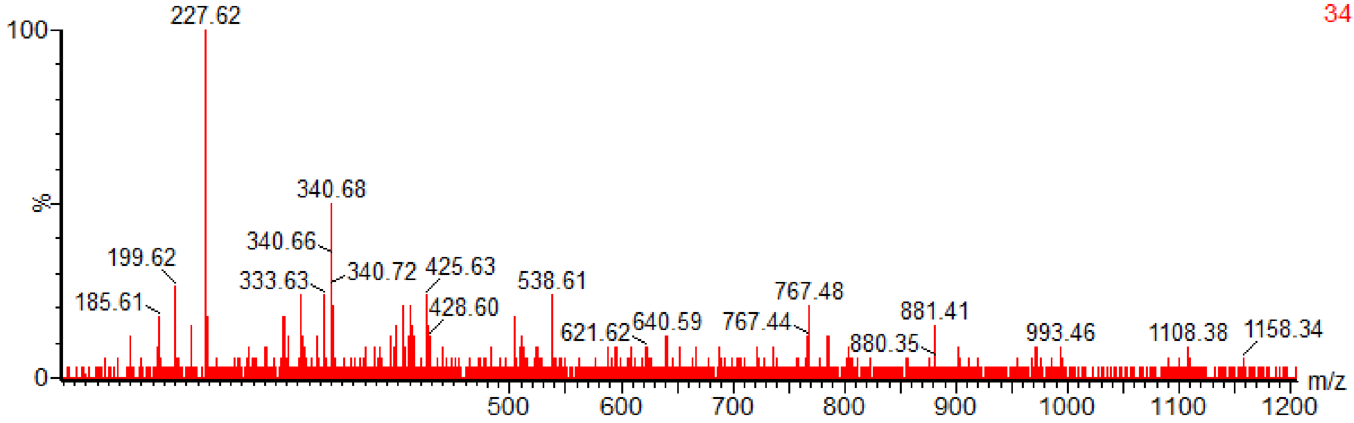

KGLDDFR

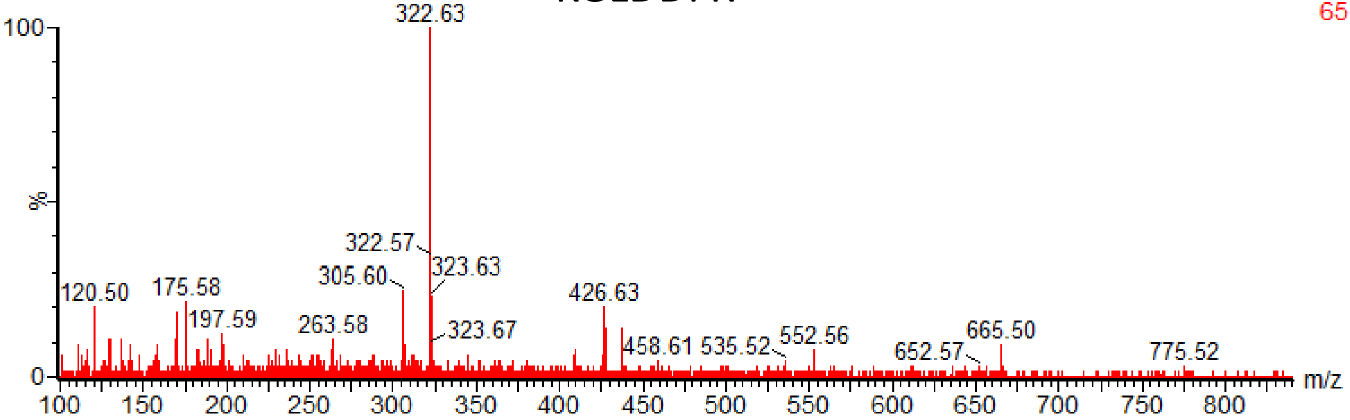

Supplemental Figure 1

MSMS spectra for the proteins identified by one peptide
Experiment 1, undifferentiated cells

GKLDGNQDLIR

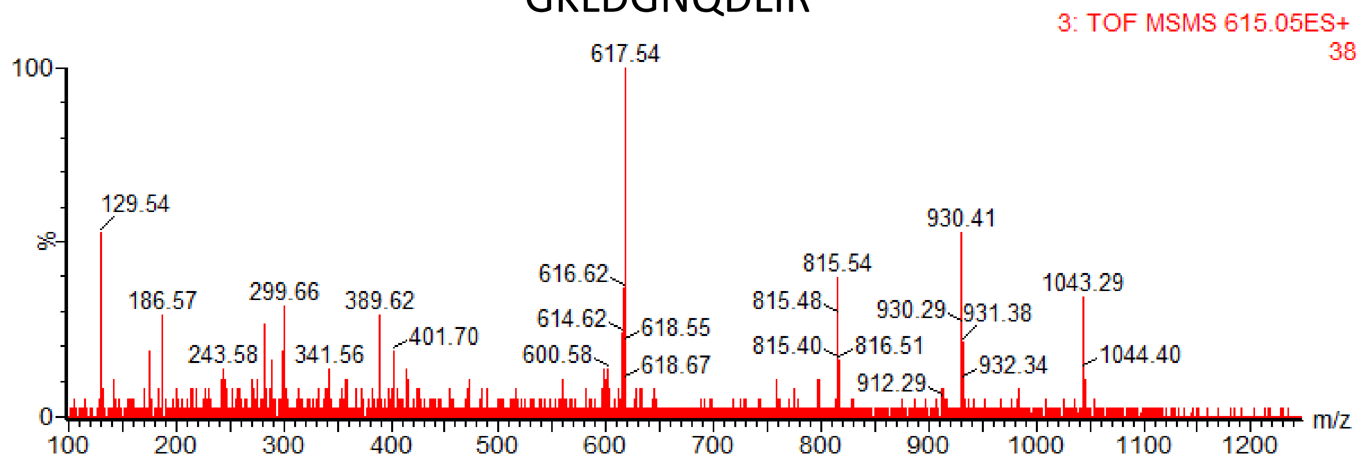

TSRPENAIIYNNNEDFQVGQAK

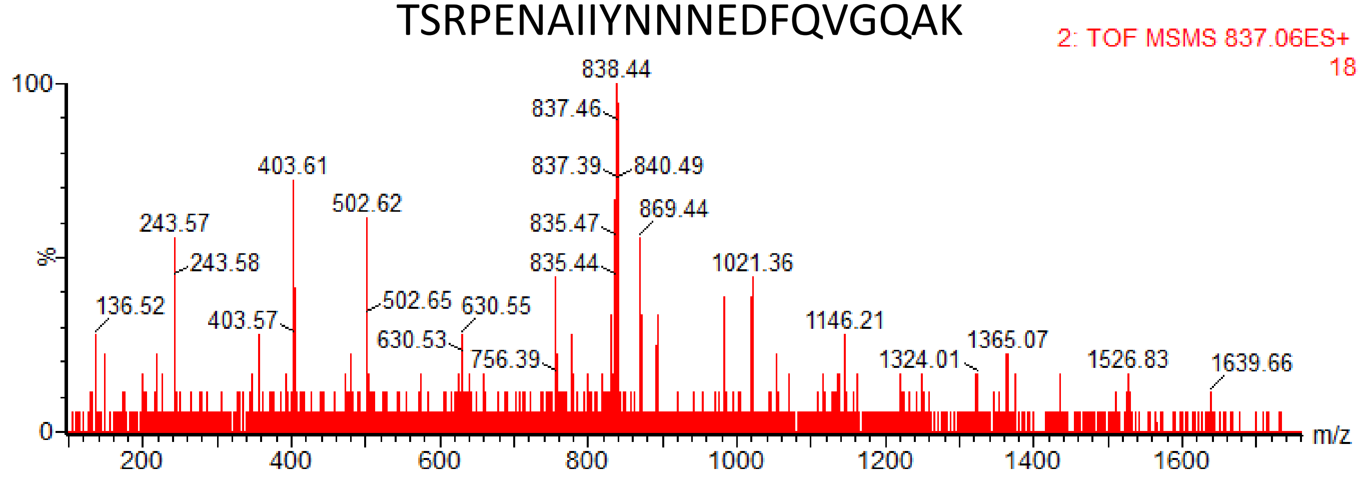

ALLSYDGLNQR

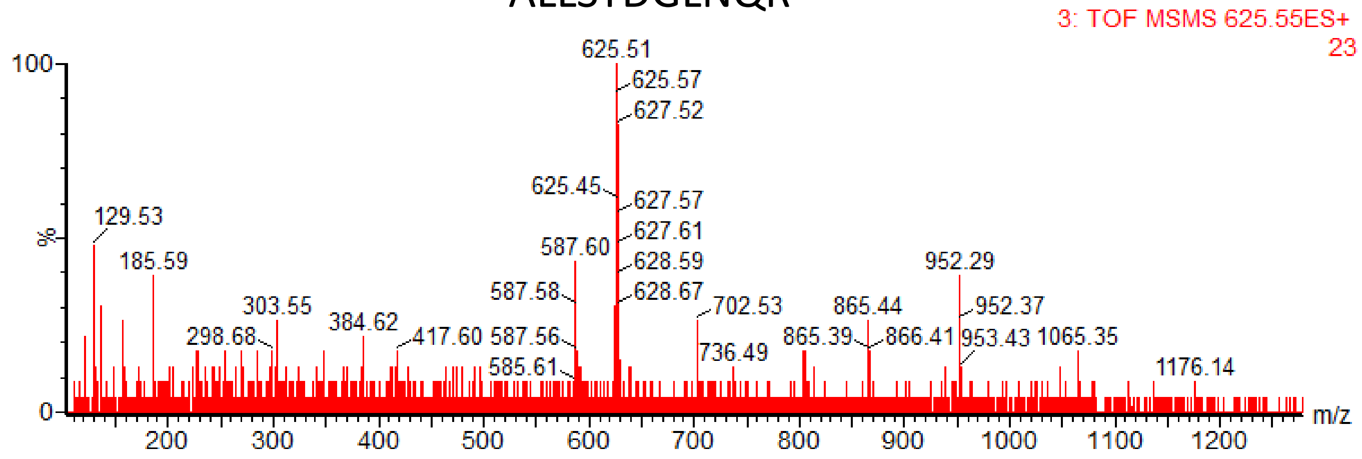

Supplemental Figure 1

MSMS spectra for the proteins identified by one peptide
Experiment 1, undifferentiated cells

LVIPSELGYGER

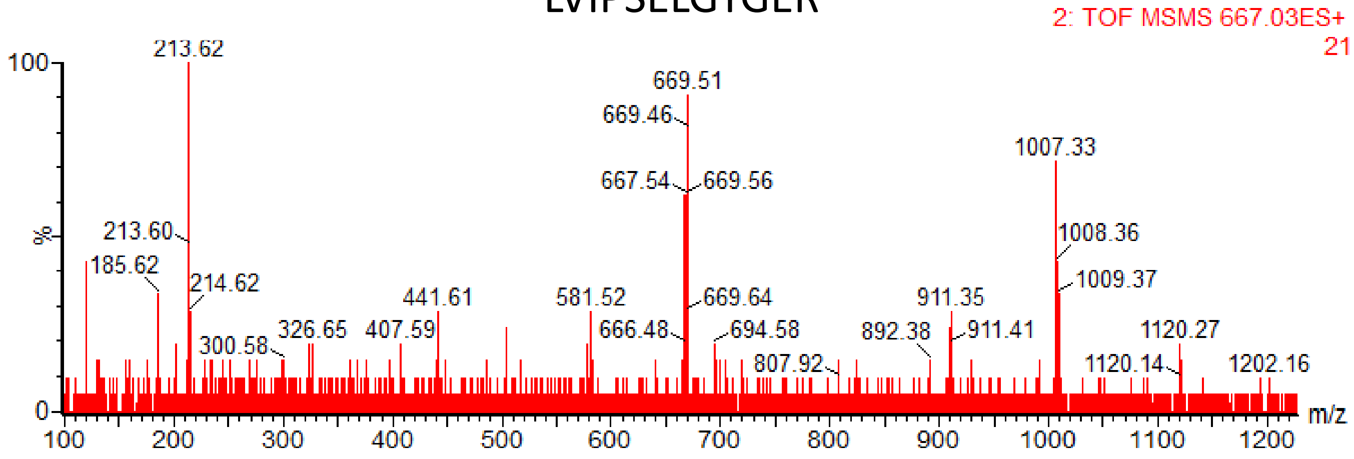

QITVNDLPVGR

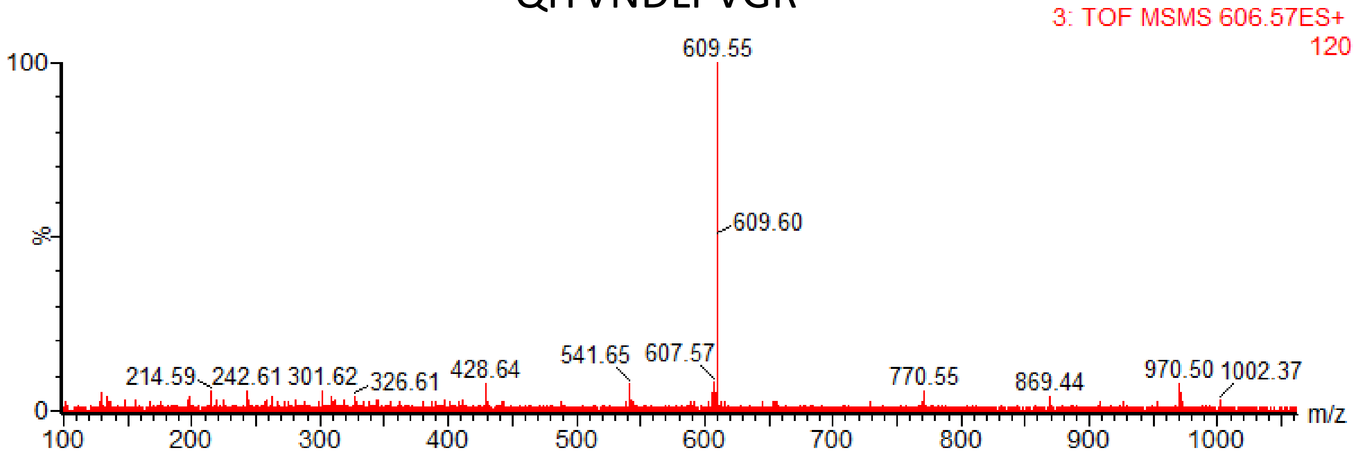

Supplemental Figure 1

MSMS spectra for the proteins identified by one peptide
Experiment 1, differentiated cells

AQFEGIVTDLIR

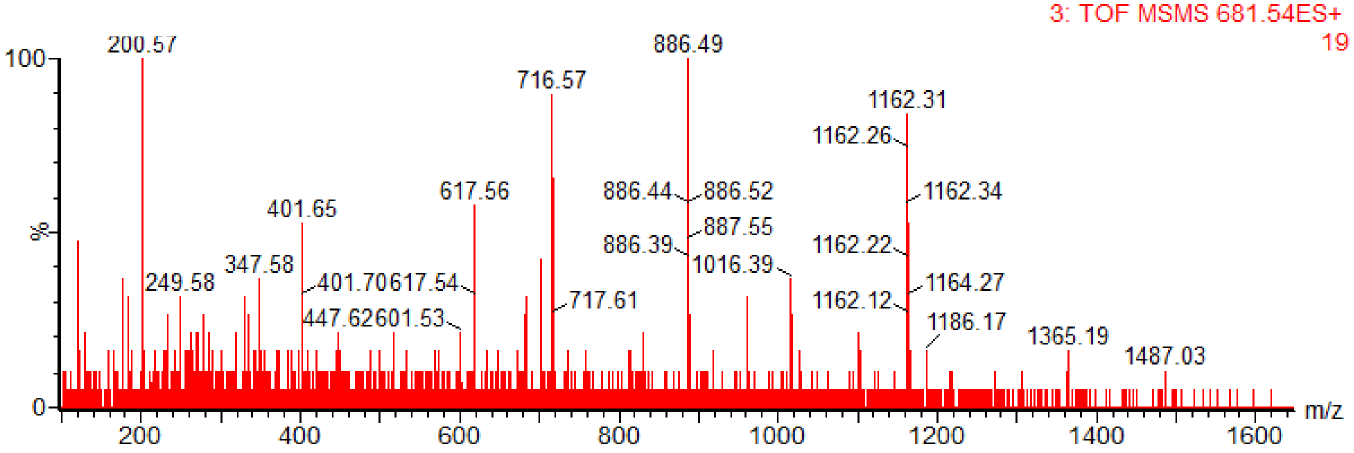

SVTEQGAELSNEER

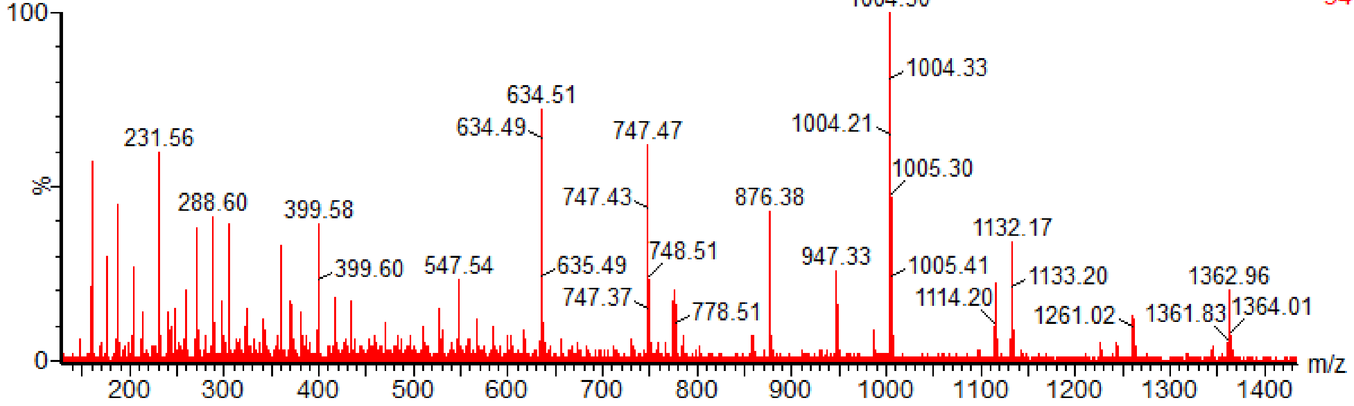

VGVDGFGR

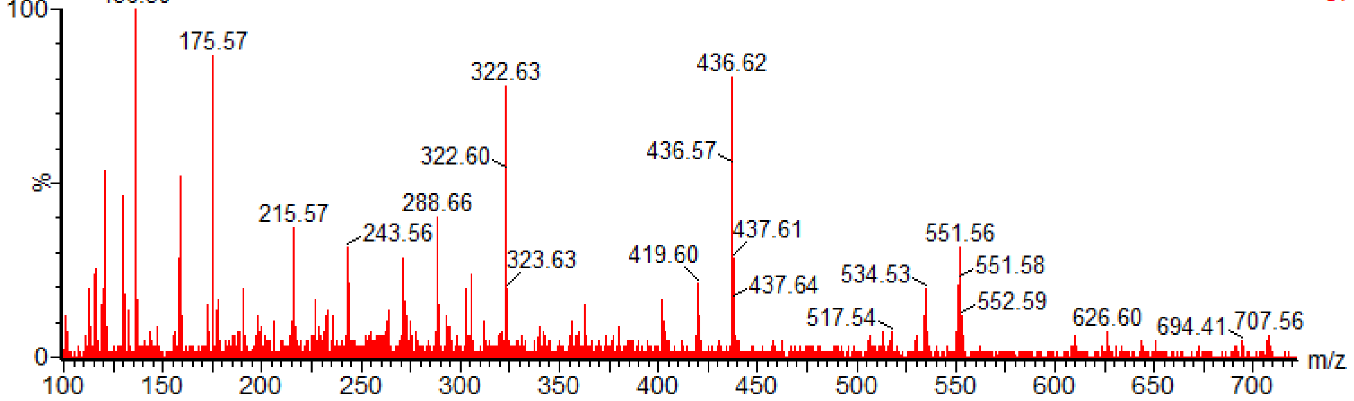

Supplemental Figure 1

MSMS spectra for the proteins identified by one peptide
Experiment 1, differentiated cells

EALGGMNSR

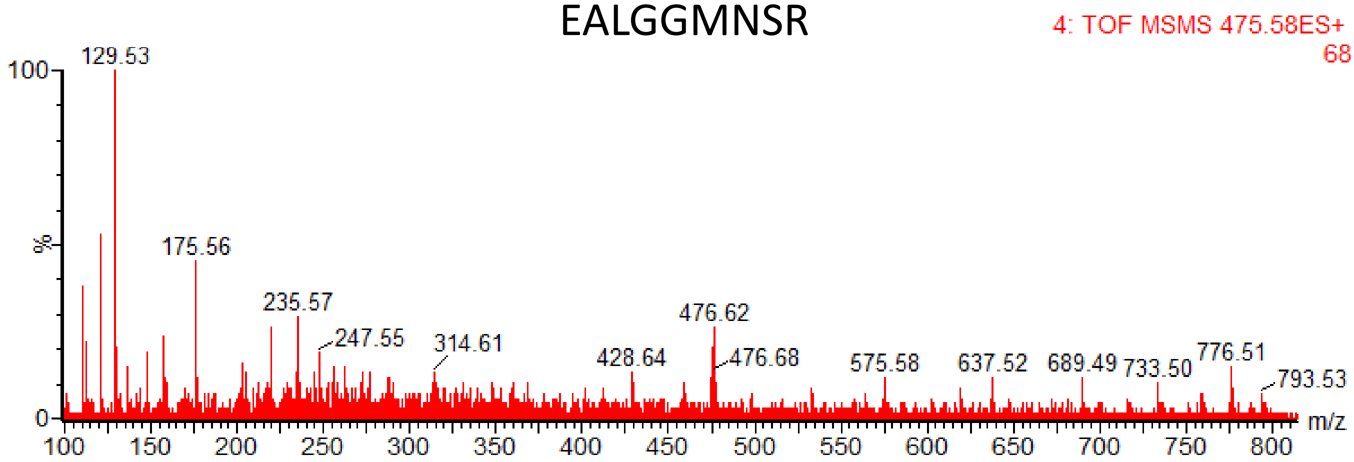

NPELQNLLLDDFFK

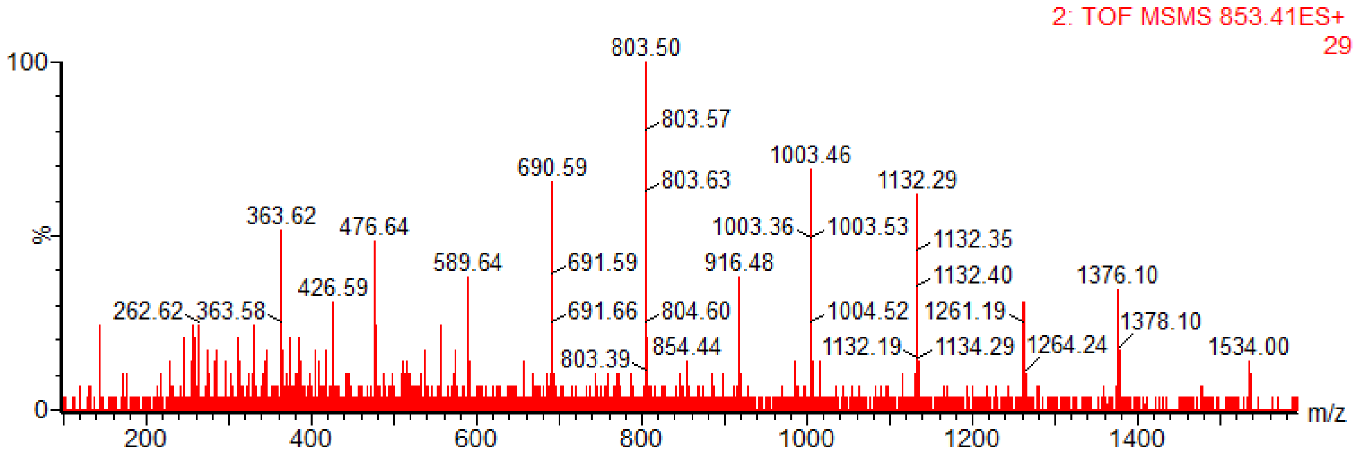

KEPEAFDWSPVVTYVCDLEGNR

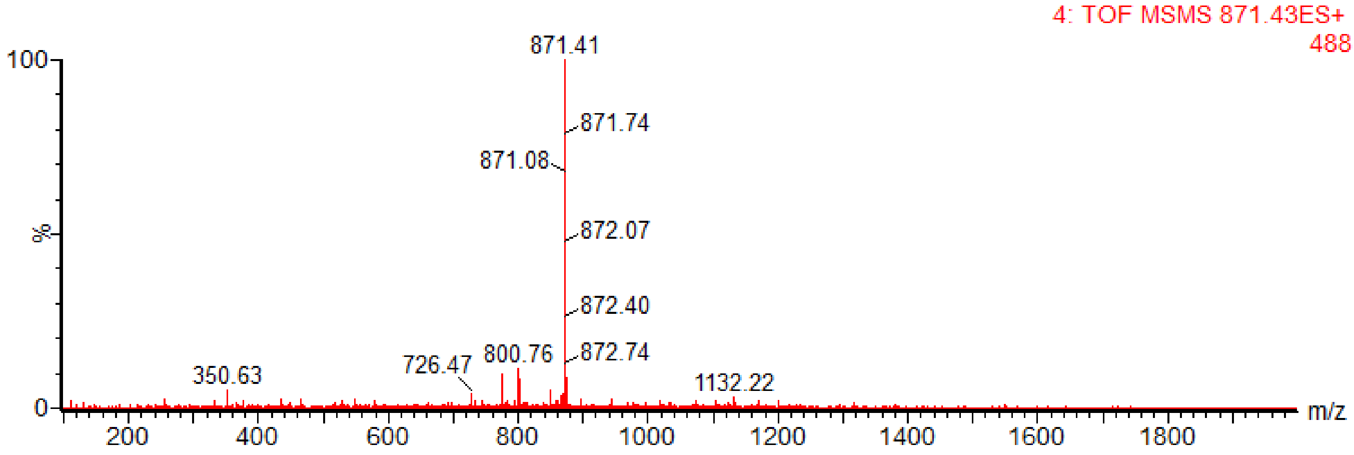

Supplemental Figure 1

MSMS spectra for the proteins identified by one peptide
Experiment 1, differentiated cells

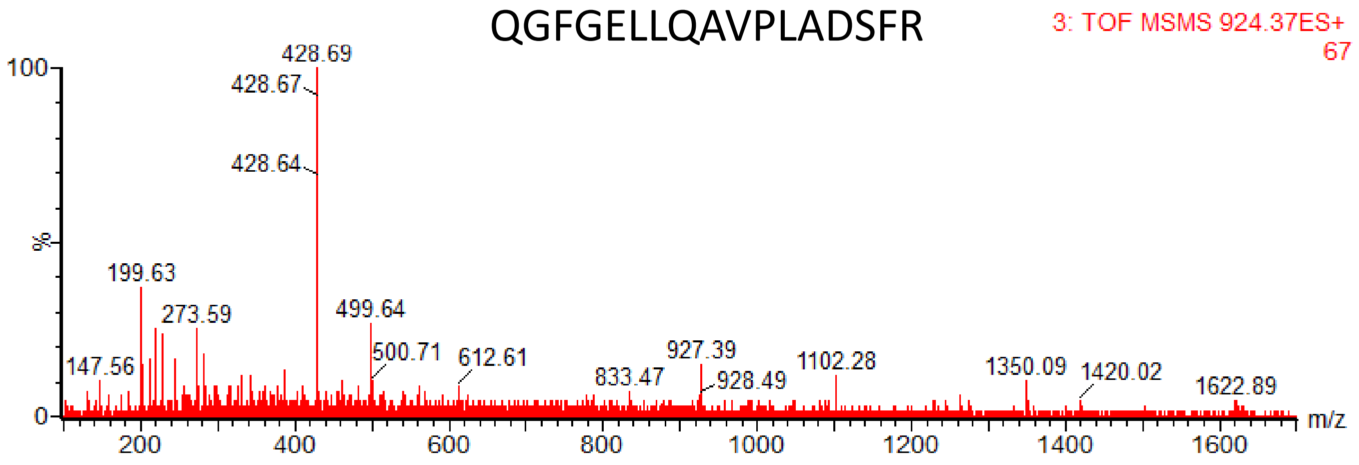

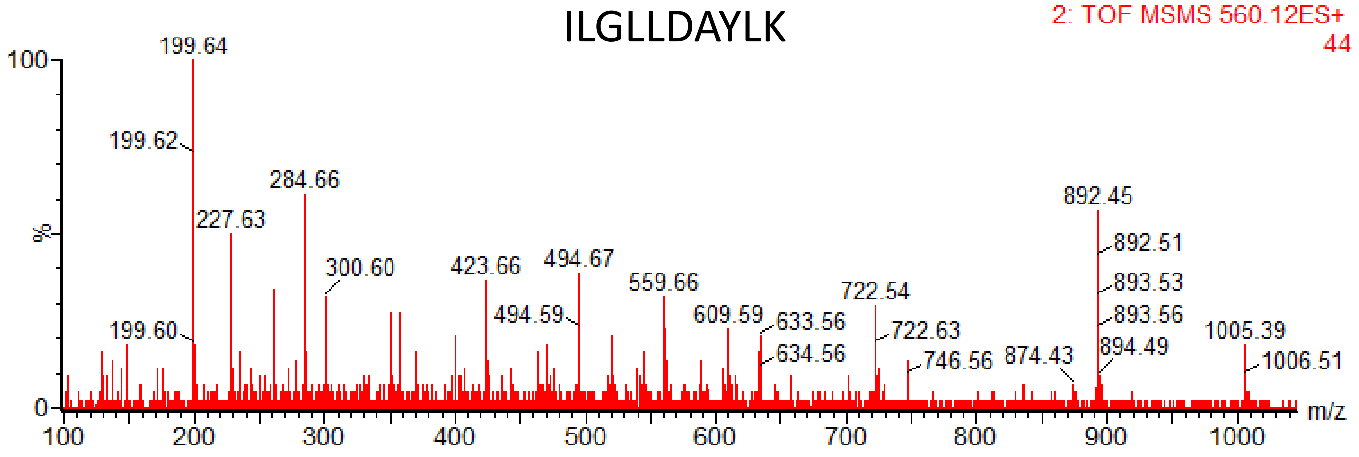

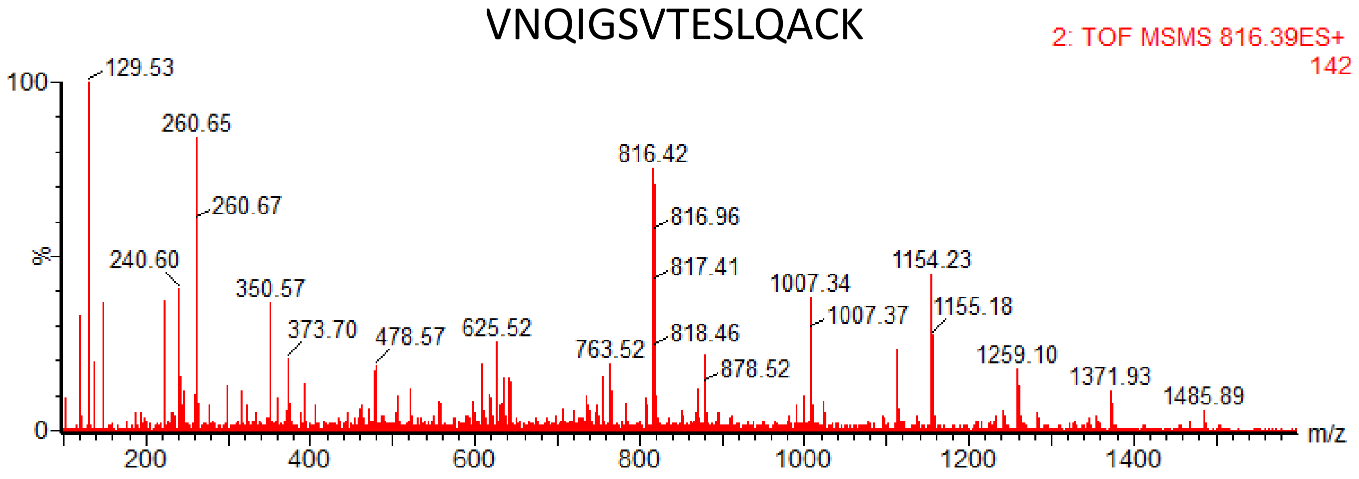

Supplemental Figure 1

MSMS spectra for the proteins identified by one peptide
Experiment 1, differentiated cells

YALYDATYETK

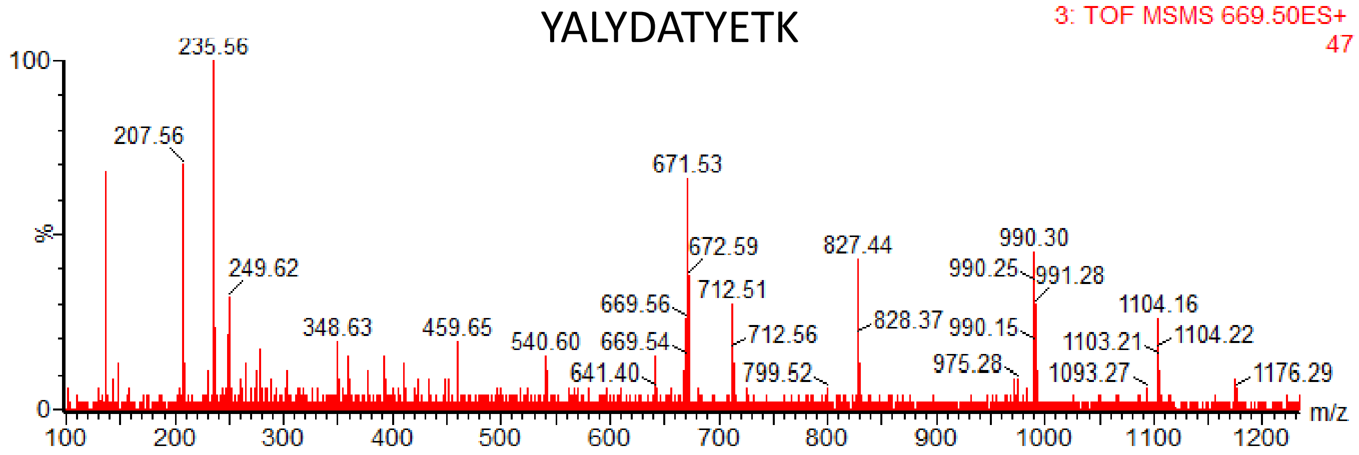

HLSVNDLPVGR

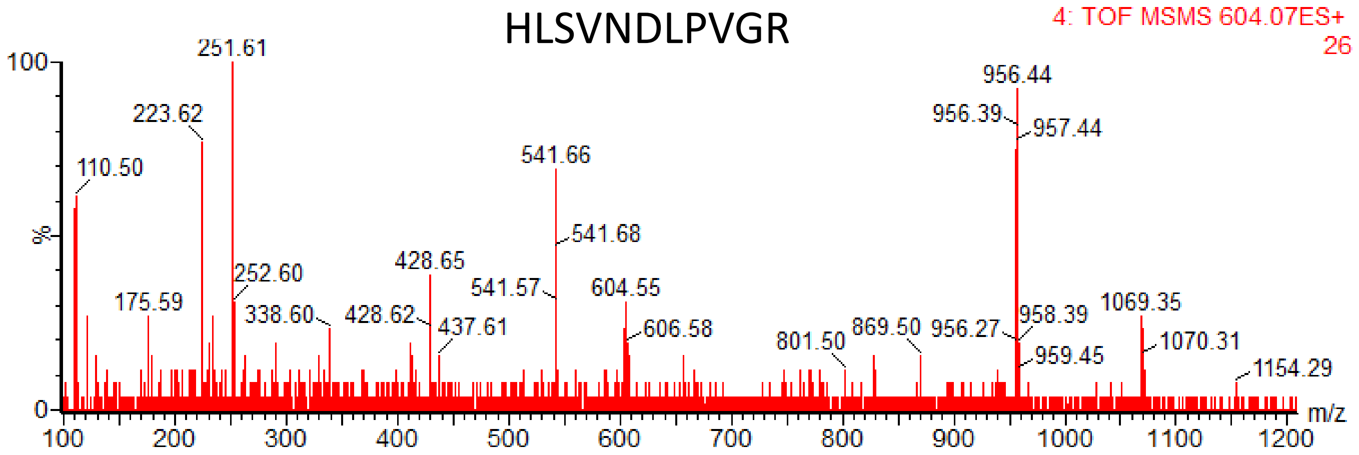

SFPDFPTPGVVFR

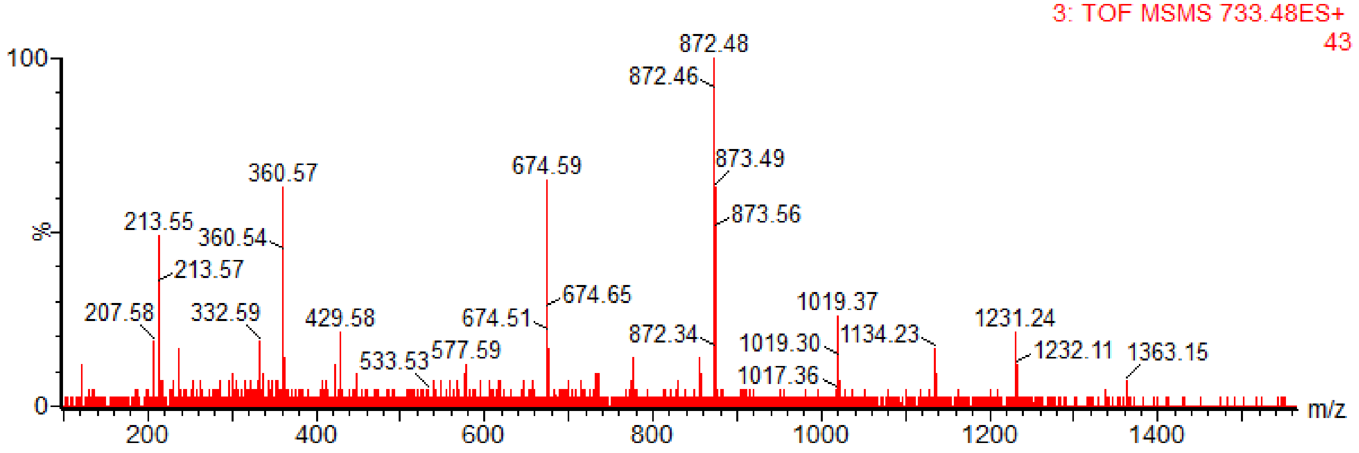

Supplemental Figure 1

MSMS spectra for the proteins identified by one peptide
Experiment 1, undifferentiated cells

FAGDKGYLTK

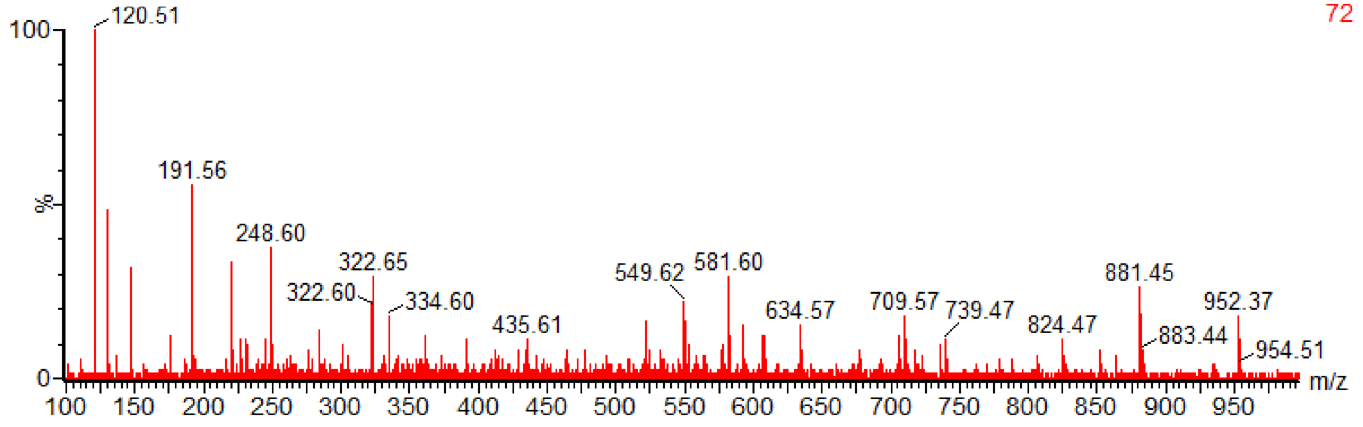

Supplemental Figure 1

MSMS spectra for the proteins identified by one peptide
Experiment 2, undifferentiated cells

ITPSYVAFTPEGER

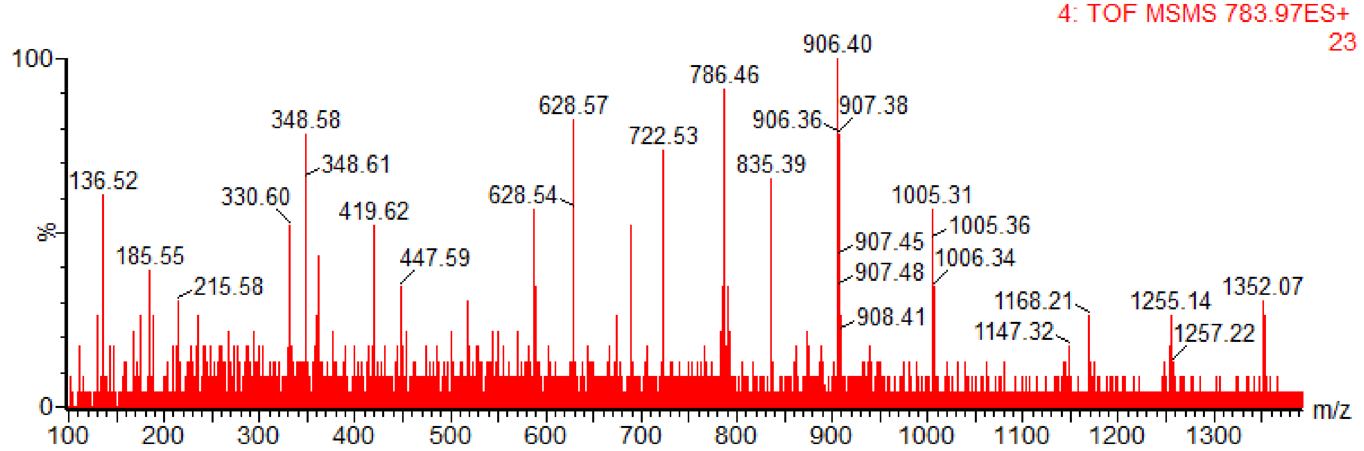

NFEDVAFDEKK

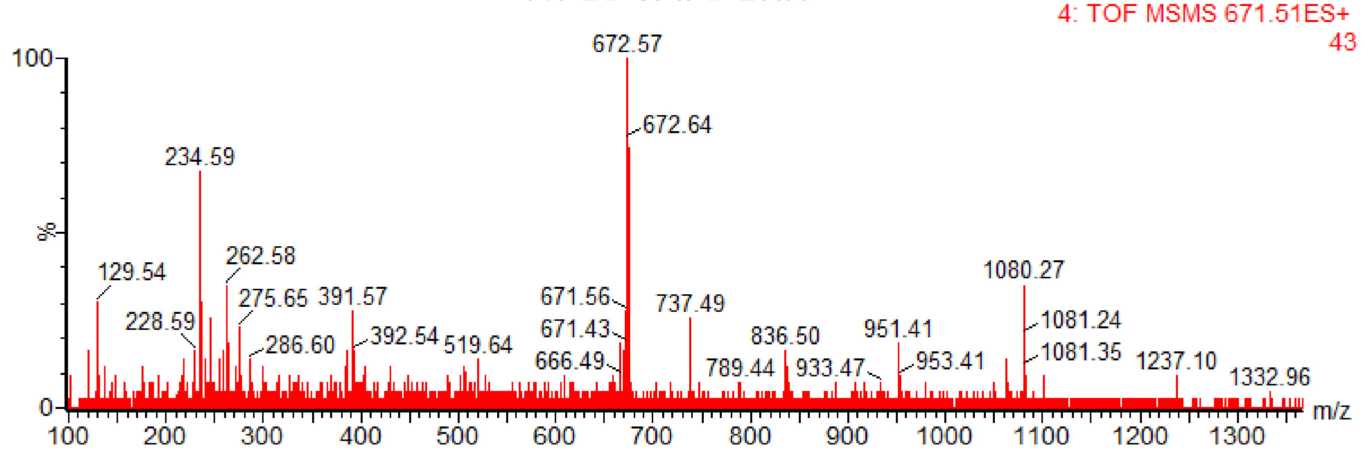

Supplemental Figure 1

MSMS spectra for the proteins identified by one peptide
Experiment 2, differentiated cells

ISGSILNELIGLVR

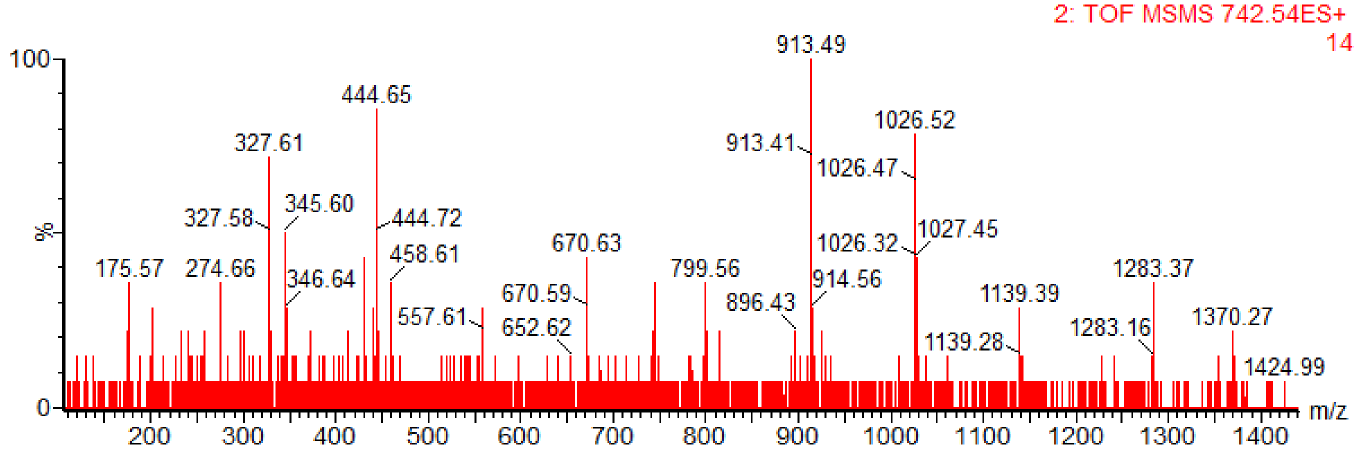

LVIDQIDNGFFSPK

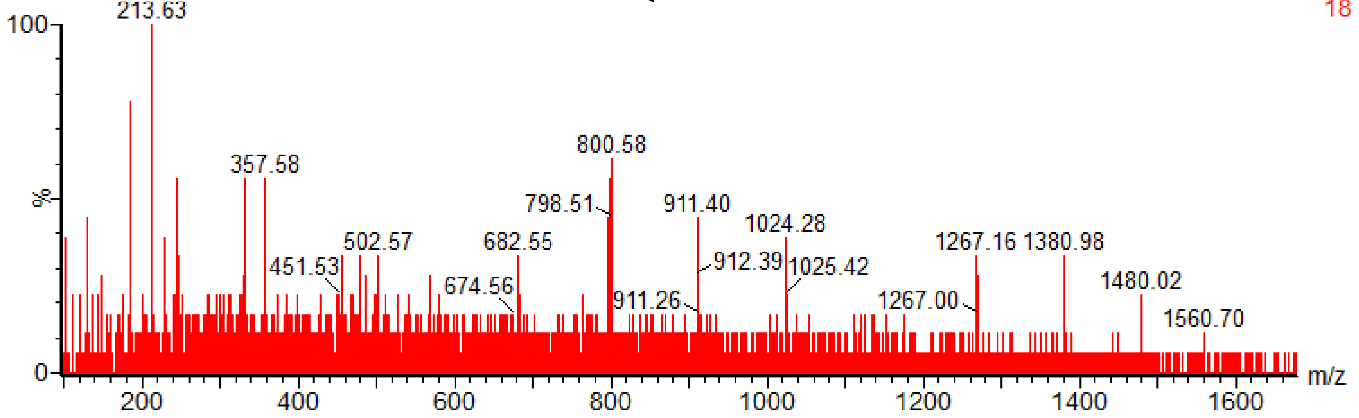

ILTFDQLALDSPK

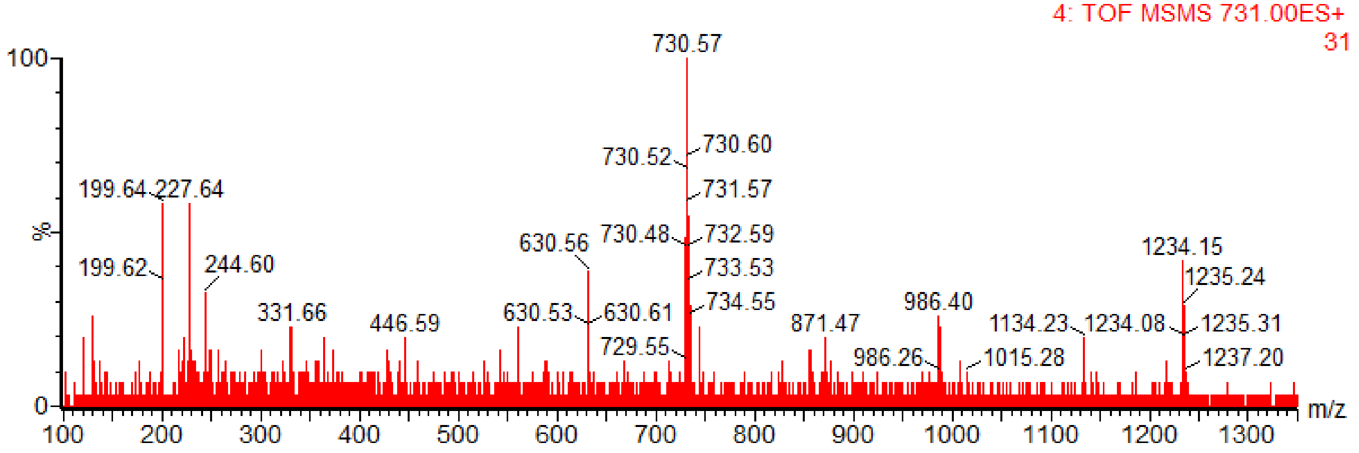

Supplemental Figure 1

MSMS spectra for the proteins identified by one peptide
Experiment 2, differentiated cells

SPAGLQVLNDYLADK

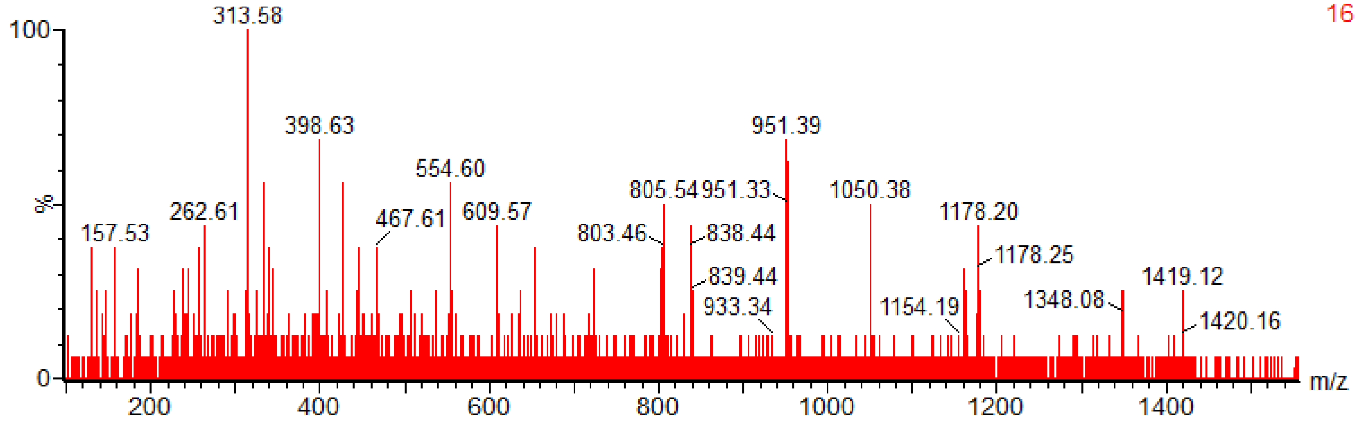

NDLSPTTVMSEGAR

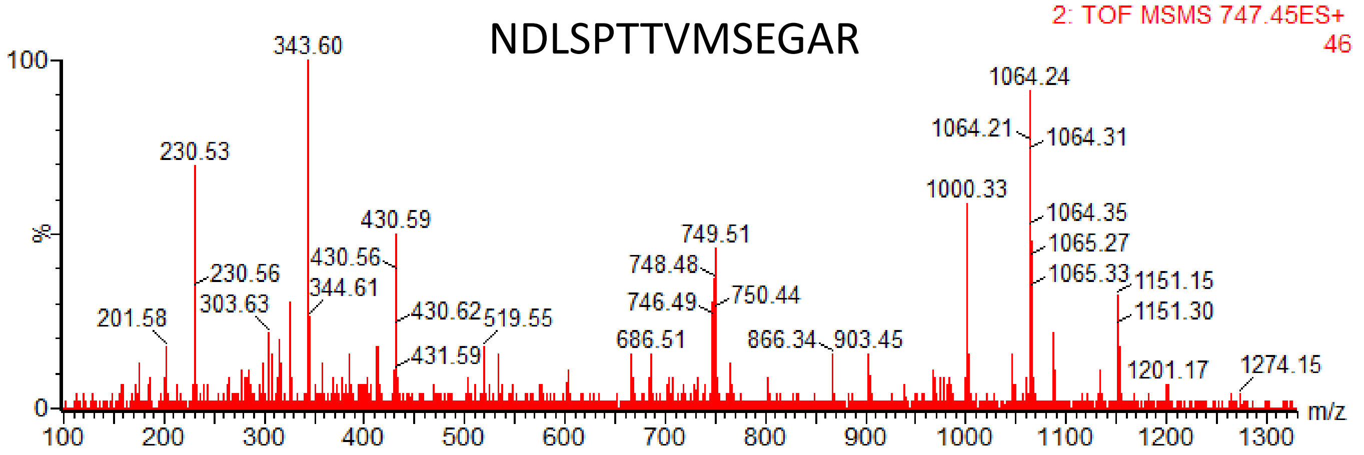

NEPQNATGAPGR

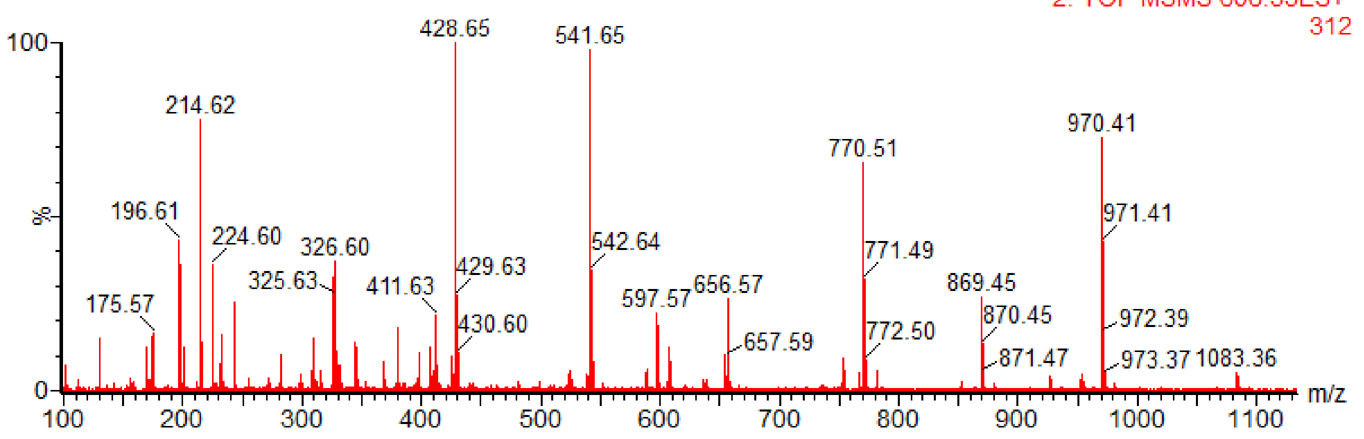

Supplemental Figure 1

MSMS spectra for the proteins identified by one peptide
Experiment 2, differentiated cells

VPSLVGSFIR

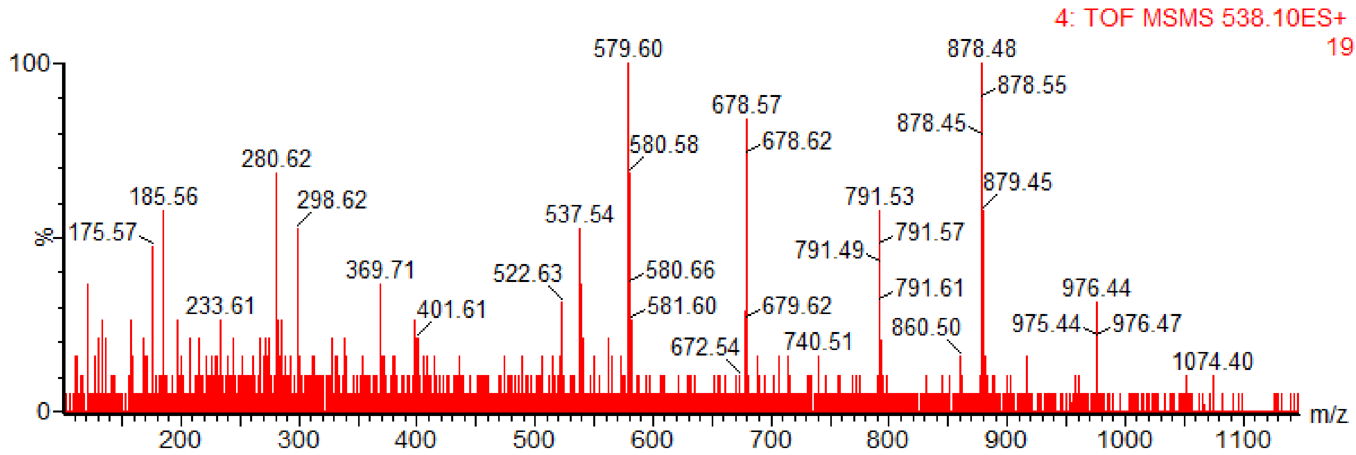

TGAAPIIDVVR

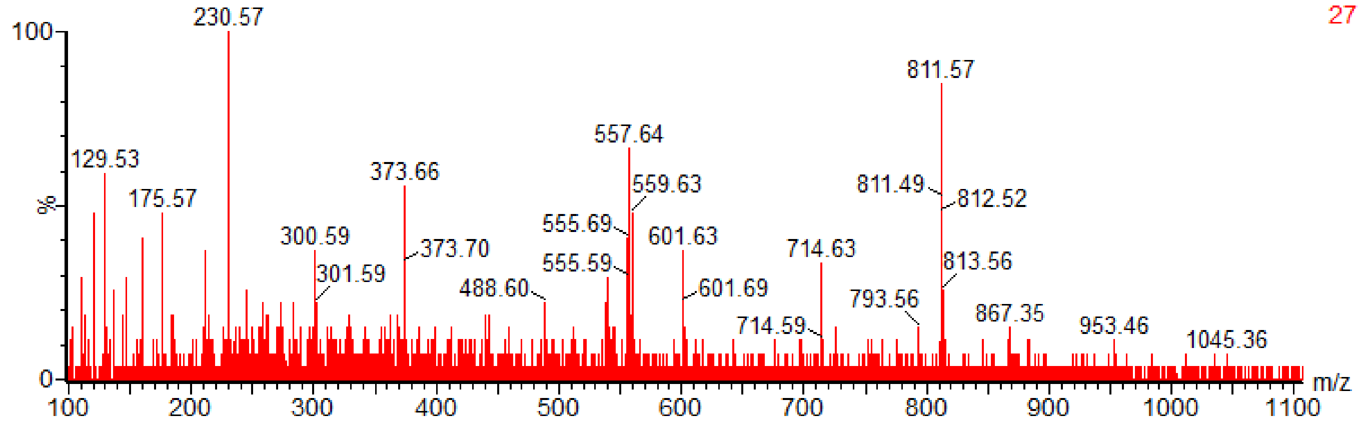

GELLEAIKR

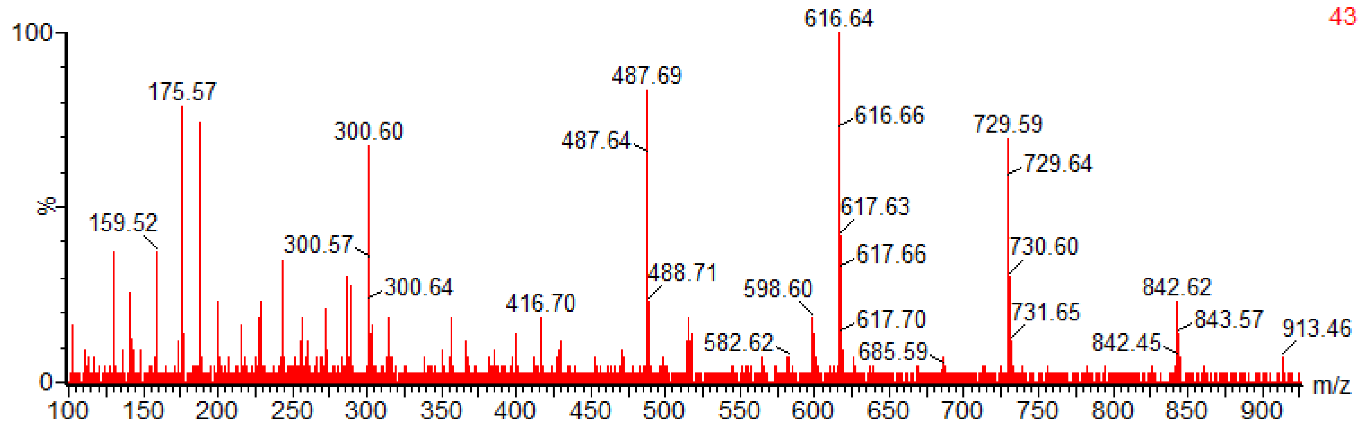

Supplemental Figure 1

MSMS spectra for the proteins identified by one peptide
Experiment 2, differentiated cells

FAGDKGYLTK

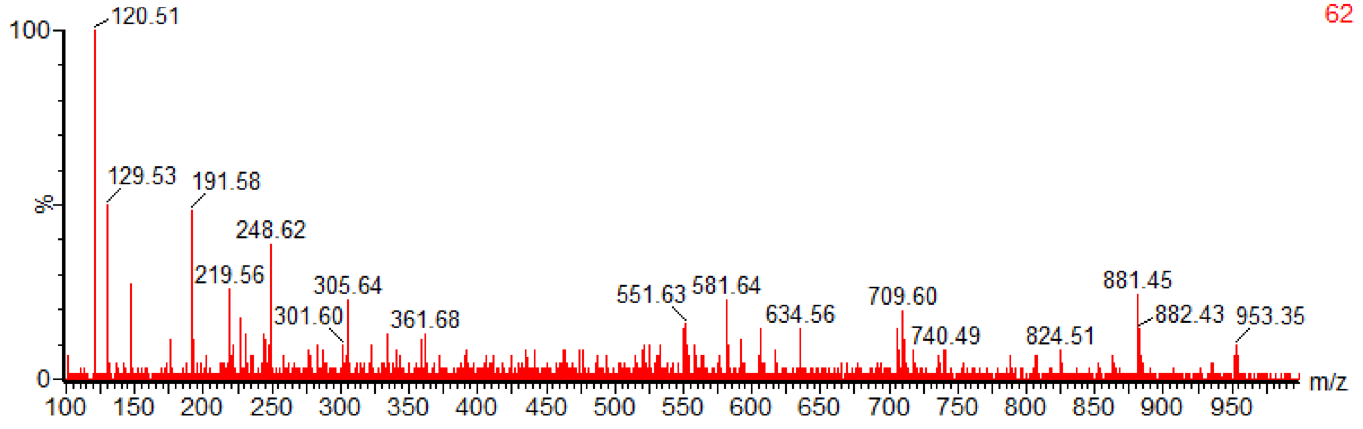

PMFIVNTNVPR

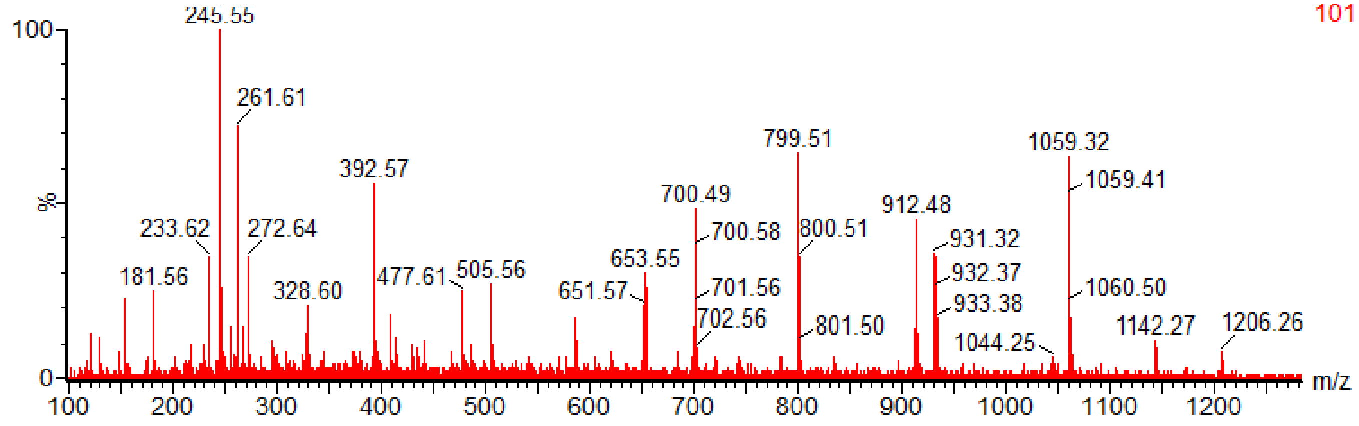

FYALSASFEPFSNK

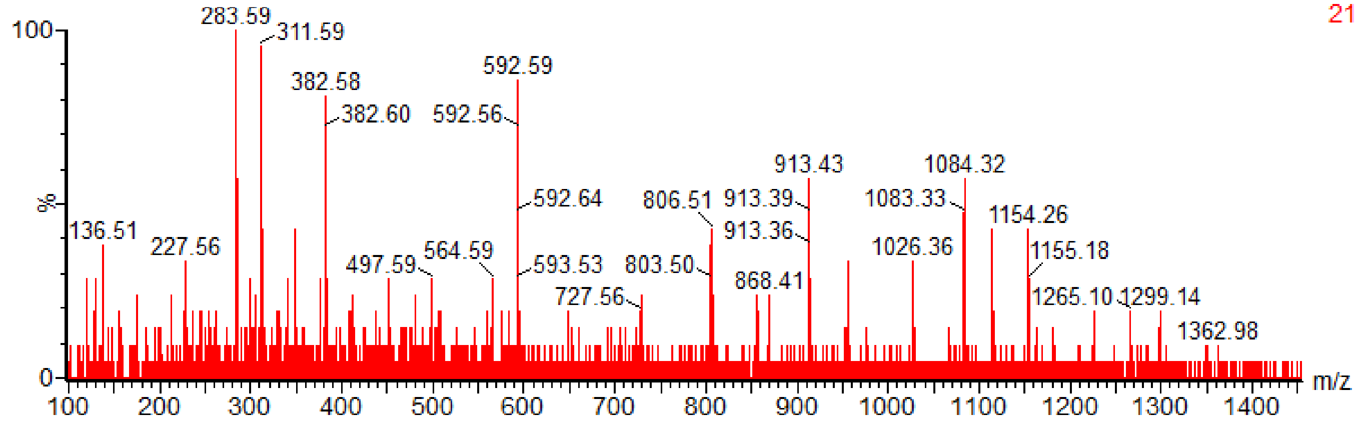

Supplemental Figure 1

MSMS spectra for the proteins identified by one peptide
Experiment 2, differentiated cells

ALVHERDEAAYGELR

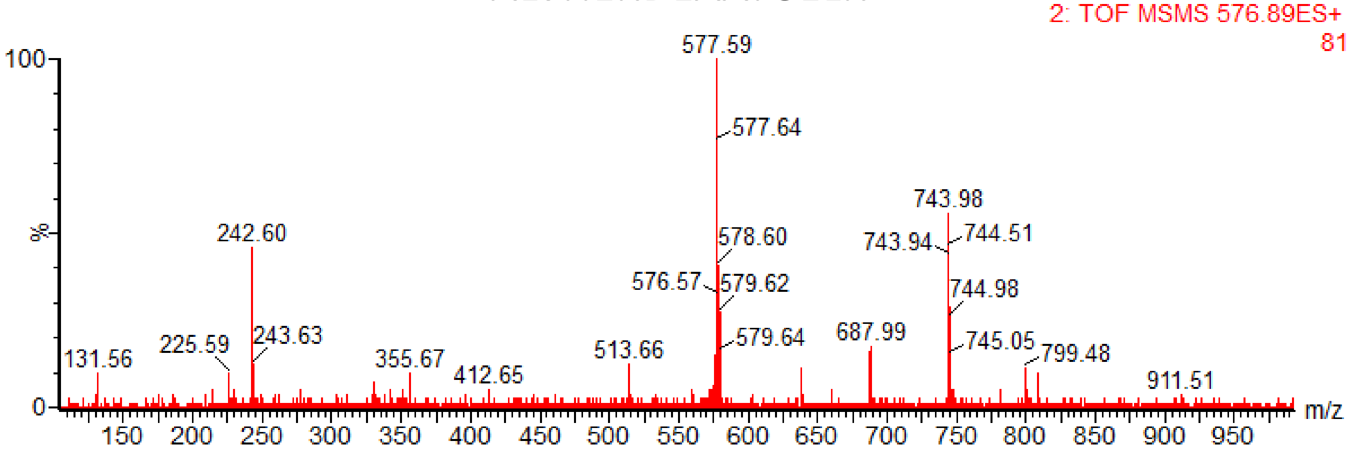

Supplement: Additional file 3 — Figure S1. MS/MS spectra that led to identification of a protein by a unique peptide. In the (+) cells, the MS/MS spectra were identified with a Mascot score of 50 or higher. In the (−) cells, all MS/MS spectra are shown. [file 1477-5956-10-47-S3.pdf]
